# Using CRISPR-Cas9-mediated genome editing to generate *C*. *difficile* mutants defective in selenoproteins synthesis

**DOI:** 10.1038/s41598-017-15236-5

**Published:** 2017-11-07

**Authors:** Kathleen N. McAllister, Laurent Bouillaut, Jennifer N. Kahn, William T. Self, Joseph A. Sorg

**Affiliations:** 1 Department of Biology, Texas A&M University, College Station, TX USA; 2 Department of Molecular Biology & Microbiology, Tufts University School of Medicine, Boston, MA USA; 3 Burnett School of Biomedical Sciences, University of Central Florida, Orlando, FL USA; 4 Present Address: Matrivax R&D Corp. 650 Albany Street, Boston, MA USA

## Abstract

*Clostridium difficile* is a significant concern as a nosocomial pathogen, and genetic tools are important when analyzing the physiology of such organisms so that the underlying physiology/pathogenesis of the organisms can be studied. Here, we used TargeTron to investigate the role of selenoproteins in *C*. *difficile* Stickland metabolism and found that a TargeTron insertion into *selD*, encoding the selenophosphate synthetase that is essential for the specific incorporation of selenium into selenoproteins, results in a significant growth defect and a global loss of selenium incorporation. However, because of potential polar effects of the TargeTron insertion, we developed a CRISPR-Cas9 mutagenesis system for *C*. *difficile*. This system rapidly and efficiently introduces site-specific mutations into the *C*. *difficile* genome (20–50% mutation frequency). The *selD* CRISPR deletion mutant had a growth defect in protein-rich medium and mimicked the phenotype of a generated TargeTron *selD* mutation. Our findings suggest that Stickland metabolism could be a target for future antibiotic therapies and that the CRISPR-Cas9 system can introduce rapid and efficient modifications into the *C*. *difficile* genome.

## Introduction

*Clostridioides difficile*, more commonly known as *Clostridium difficile* ^1,2^, is a Gram-positive, anaerobic, spore-forming bacterium that is the major cause of antibiotic-associated diarrhea. Most-commonly, patients undergoing antibiotic treatment are at high risk for *C*. *difficile* infection (CDI) due to the disruption of the normal colonic microbiota by broad-spectrum antibiotics^3–5^. Spores, the metabolically-dormant form of *C*. *difficile* that can survive passage between hosts in the aerobic environment, are ingested by a host and germinate in response to small-molecule germinants (*i*.*e*., cholic acid, and its derivatives, and an amino acid, such as glycine) into a vegetative, toxin-producing cell^6–8^. These toxins, TcdA and TcdB, damage epithelial cells which results in the symptoms of CDI^9–11^.

Much of our understanding of *C*. *difficile* physiology has come in the last few years and coincided with the development of genetic tools for this organism^12–17^. The tools available to genetically manipulate *C*. *difficile* include: i) single-crossover integration of segregationally unstable plasmids^15,16^; ii) mobile, group II introns (TargeTron/ClosTron technology)^14^; iii) Allelic-Coupled Exchange using either the *codA* or *pyrE* systems^12,13^; and iv) Mariner transposition^17,18^. To date, the most widely used system is the TargeTron (or ClosTron) system which relies on the re-targeting of mobilizable group II introns and the use of retrotransposable activated markers (RAM)^19^. Though RAM markers allow for the easy identification of potential mutants, unfortunately, this system only creates insertion mutations resulting in potential polar effects on downstream genes. In addition, the inserted antibiotic resistance marker has led to perceived hypervirulence of mutant strains in the hamster model of CDI^20,21^.

Segregationally unstable plasmids can be used to create single insertions into the *C*. *difficile* genome^15,16^ or can be used as allelic exchange plasmids using *codA* counter selection or 5-fluoroorotic acid (FOA) in a pre-generated *pyrE* mutant^12,13^. Due to the segregationally unstable nature of the plasmids, daughter cells may not receive a copy of the plasmid and are killed by the antibiotic in the surrounding medium. Strains with single-integration events of the plasmid, due to homologous recombination, grow more rapidly. These ‘large’ colonies are then spread on nutrient-poor medium supplemented with 5-fluorocytosine (for *codA-*based plasmids) or FOA for the *pyrE* allelic exchange system^12,13^. Due to nutrient-poor media being a requirement for counter-selecting the integrated plasmids, mutations that generate slow-growth phenotypes or loss of metabolic pathways may result in difficulties in growth on such medium. Moreover, the *pyrE* system requires the correction of the pre-generated *pyrE* mutation before progressing experimentally – increasing the effort and time required to generate mutant strains^13^. On the other hand, advantages to this system are the ability to create single nucleotide mutations and clean deletions and the ability to integrate a single, chromosomal copy for complementation.

In a previous study, the TargeTron system was used to create insertions in genes (*i*.*e*., *prdR*, *prdB* and *grdA*) whose products are involved in Stickland metabolism^22^. Stickland reactions are a primary source of energy for a small group of anaerobic bacteria grown that typically use amino acids as their sole carbon and nitrogen sources^23^. During these reactions, one amino acid, such as alanine or leucine, is oxidatively decarboxylated or deaminated^23^. Subsequently, in the reductive branch, D-proline or glycine acts as electron acceptors. In the case of D-proline the amino acid ring is reduced and converted to δ-amino valeric acid^24^. In the case of glycine reductase, two selenoproteins are involved (GrdB and GrdA) and the glycine is deaminated in a process that results in acetyl-phosphate (and thus ATP) production. PrdB, GrdA and GrdB are the selenium-containing subunits of the respective reductases and their expression is regulated by PrdR^22^. These enzymes are important for growth in protein-rich medium supplemented with proline or glycine indicating that selenium-containing enzymes are important for *C*. *difficile* physiology^22^.

Selenophosphate synthetase (SelD, CDR20291_2388) has been shown to be necessary for the activation of selenium for specific incorporation into biological macromolecules in several bacterial model systems^25^. Selenium incorporation in *C*. *difficile* is likely to be dependent on a selenophosphate synthetase (SelD) which generates selenophosphate from selenide and inorganic phosphate that is incorporated into a serine-charged tRNA by selenocysteine synthase (SelA, CDR20291_2387)^26^. The selenocysteine is then incorporated into proteins such as PrdB and GrdA during translation with the aid of a selenocysteine-specific elongation factor, SelB (CDR20291_2386)^26^. SelD, SelA and SelB are encoded in a single genetic locus and likely are part of the same transcriptional unit. Selenium is used for other enzymatic processes as well and requires SelD to generate selenophosphate for these processes^25^. In order to test the importance of selenium-containing factors on *C*. *difficile* growth, we engineered a mutation in *selD* using the established TargeTron gene knock out system and found that *selD* is important for *C*. *difficile* growth and incorporation of selenium into proteins. To avoid any potential polar effects of the TargeTron system, we developed a CRISPR-Cas9 mutagenesis system and used this system to engineer a *selD* in-frame deletion mutation. Our results highlight the importance of selenoproteins in *C*. *difficile* physiology and suggest that these proteins could be used as targets for future antibiotic therapies. Moreover, this newly developed *C*. *difficile* genetic system can be used to rapidly and efficiently introduce mutations into the *C*. *difficile* genome.

## Results

### Generation of a TargeTron mutation in *C*. *difficile* JIR8094 *selD*

**Figure 1:**
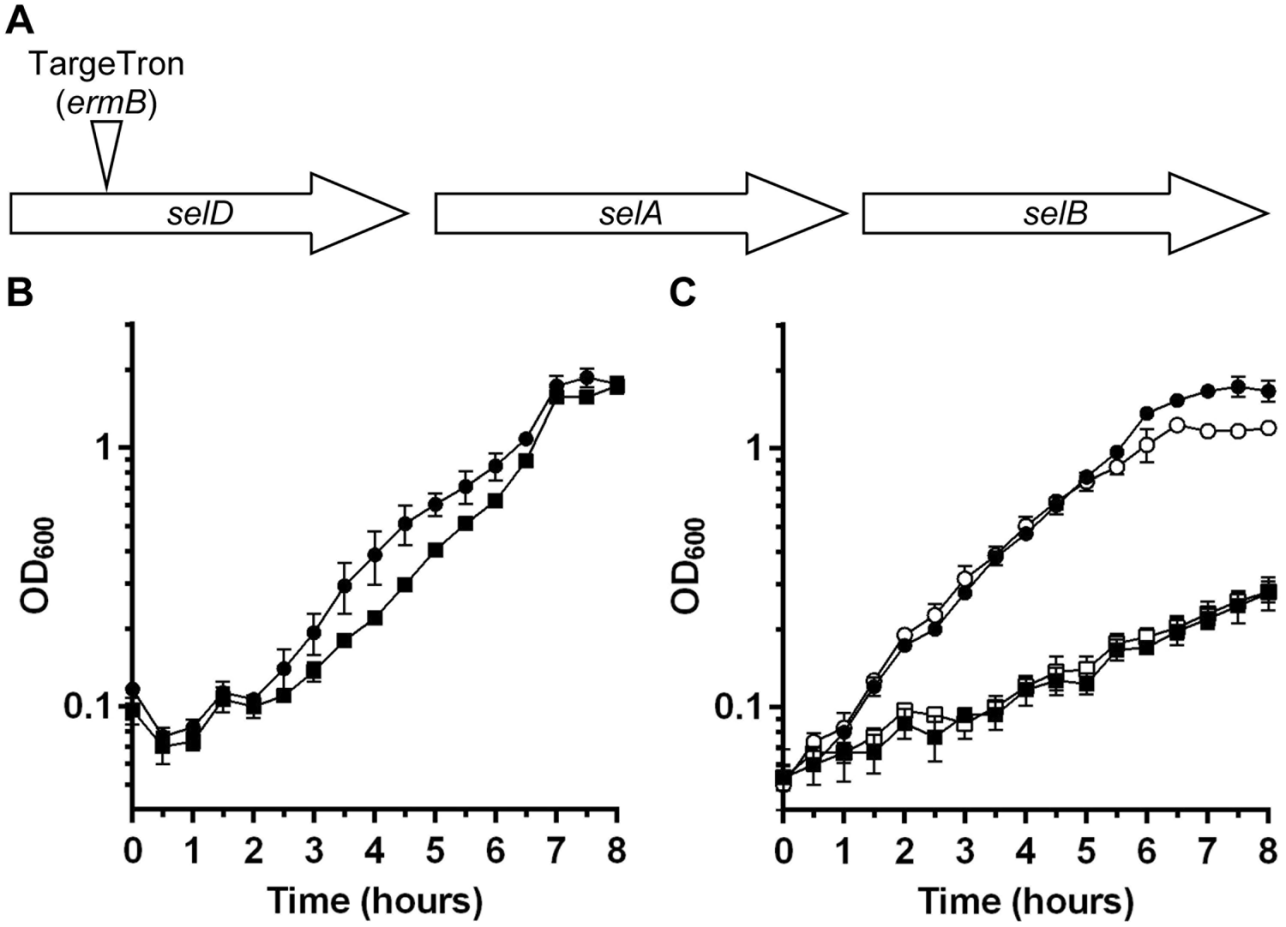
*C*. *difficile selD::ermB* has a defect in growth compared to the WT strain. (**A**) Genetic organization of the *selD* locus: *selD* (CDR20291_2388), *selA* (CDR20291_2387) and *selB* (CDR20291_2386). The location of the TargeTron insertion into *selD* is illustrated. (**B**) *C*. *difficile* JIR8094 (●) and *C*. *difficile* LB-CD7 (*selD::ermB*) (■) were grown in BHIS medium and growth was monitored over time. (**C**) *C*. *difficile* JIR8094 was grown in TYG (●) or TY (○) medium and *C*. *difficile* LB-CD7 was grown in TYG (■) or TY (□) medium and growth was monitored over time. In both graphs, data points represent the average from three independent experiments and error bars represent the standard deviation of the mean.

In order to investigate the role of selenoproteins on *C*. *difficile* physiology, we generated a TargeTron insertion into the *selD* gene of *C*. *difficile* strain JIR8094. *C*. *difficile selD* is the first gene in the operon and is upstream of the genes encoding a selenocysteine synthase (*selA*) and a selenocysteine-specific elongation factor (*selB*) (Fig. 1A). The parental strain, JIR8094, and the *selD* mutant, LB-CD7, grew to nearly equal levels in rich BHIS medium (Fig. 1B). We next tested the growth of the two strains in protein-rich conditions; tryptone is a rich source of amino nitrogen^27^ and we reasoned that this medium may favor Stickland metabolism. In either TY or TYG medium, growth of strain LB-CD7 was significantly decreased compared to that of its parent strain (Fig. 1C).

### *C*. *difficile selD::ermB* does not incorporate selenium into proteins

**Figure 2:**
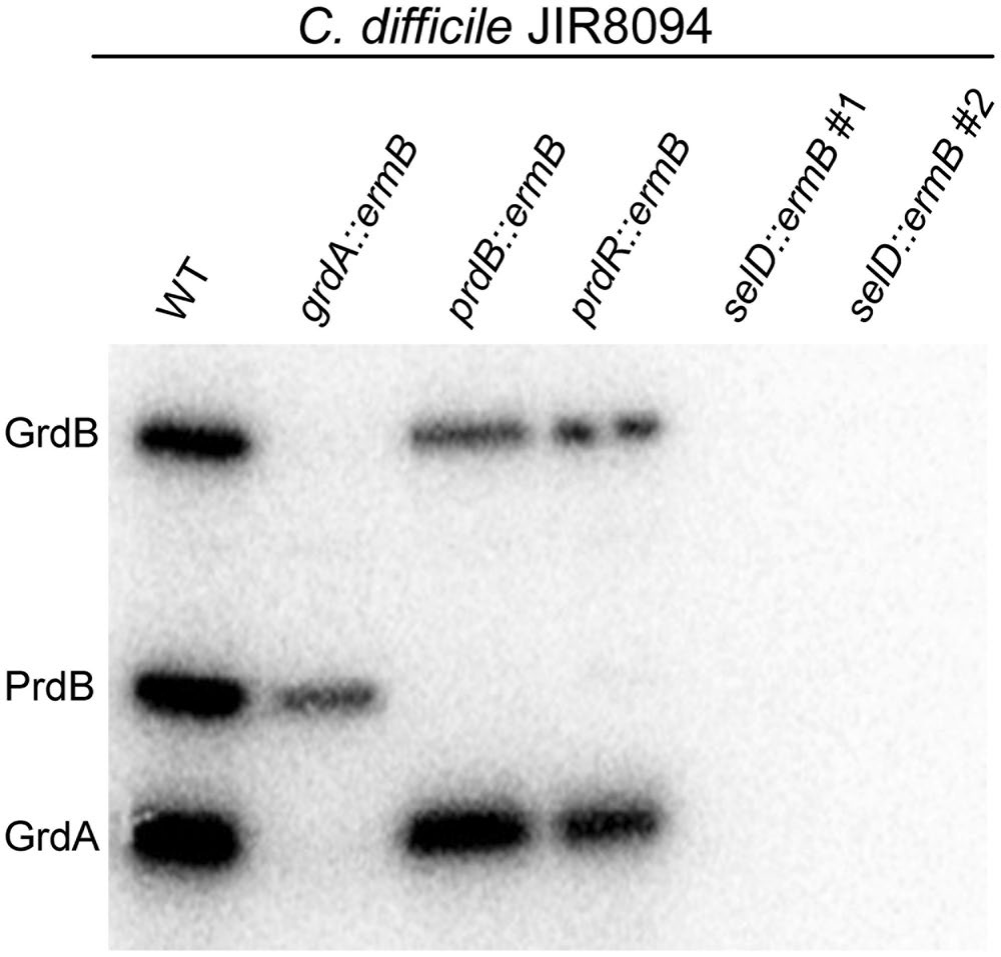
*C*. *difficile selD::ermB* does not incorporate selenium into selenoproteins *C*. *difficile* strains were grown in TY medium and 10 µCi of ^75^Se was added to the culture medium during growth. After 24 hours of growth, cultures were harvested, cells were lysed and samples from clarified lysates were separated by SDS-PAGE (15%). Radioactively-labeled protein was detected using phosphorimage analysis. GrdB, PrdB and GrdA are labeled based on previously published data^24^. The full, uncropped phosphorimage can be found in the supplemental information.

The growth defect observed for strain LB-CD7 (*selD::ermB*) suggests that selenoproteins are important for growth. To confirm that the *selD* mutation led to a loss of selenium incorporation during protein synthesis, we measured the incorporation of radioactively labeled selenium in TY medium (Fig. 2). A 1:100 dilution of overnight cultures were added to 10 mL TY medium supplemented with approximately 10 µCi of ^75^Se in the form of selenite (50 nM cold). After 24 hours cultures were harvested by centrifugation, lysed by brief sonication and clarified cell extracts were analyzed by SDS-PAGE.

When separated by SDS-PAGE and analyzed using autoradiography, we detected three distinct bands in the parental strain JIR8094 (Fig. 2). These likely correspond to GrdB (largest), PrdB and GrdA (smallest band) based on a previous study^24^. In both *prdB* and *prdR* TargeTron mutants, the PrdB band was lost; PrdR is required for *prdB* expression^28^ (Fig. 2). Similarly, in the *grdA* TargeTron mutant, both GrdA and GrdB proteins are not present; due to the insertion of the group II intron into *grdA* there were polar effects on *grdB*. Significantly, in two separate *selD::ermB* isolates (LB-CD7), we observed a lack of radioactive signal, suggesting that this mutation prevents the incorporation of selenium into all three of these established proteins thereby limiting optimal growth (Fig. 2)^24^.

### Generation of a CRISPR-Cas9 mutagenesis system for use in *C*. *difficile*

**Figure 3:**
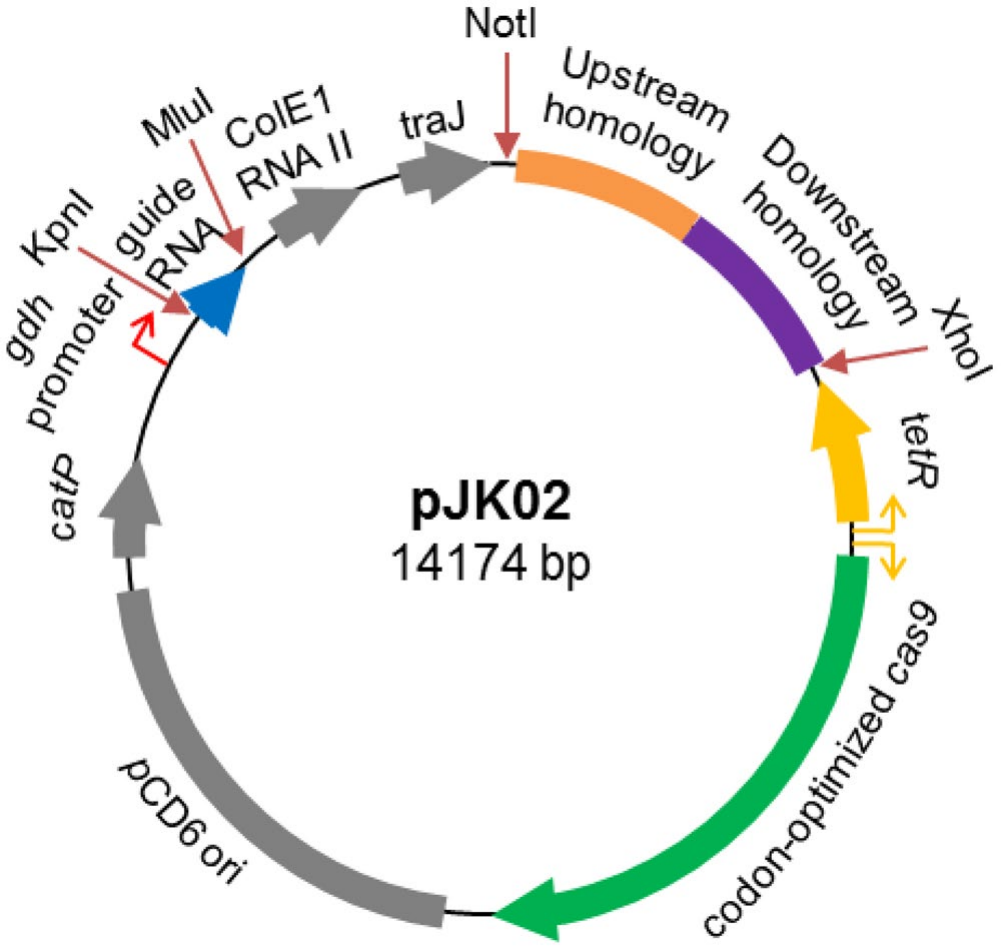
*C*. *difficile* CRISPR-Cas9 plasmid map. The pMTL84151 backbone, depicted in gray, consists of the pCD6 *C*. *difficile* (Gram-positive) replicon *oriV*, *orfB* and *repA*, the thiamphenicol resistance marker *catP*, the Gram-negative replicon *colE1*, and *traJ* for conjugal transfer from *E*. *coli*. The modifications to the backbone, depicted in various colors, consists of the insertions of the targeting region for homologous recombination, sgRNA under the expression of the *gdh* promoter, and the *tetR* promoter that regulates the expression of the *S*. *pyogenes cas9* gene that was codon optimized for expression in *C*. *difficile*.

Because the generated TargeTron insertion into *C*. *difficile selD* is likely polar on the downstream genes (*selA* and *selB*), we sought to generate a non-polar deletion in *selD* to confirm its growth disadvantage in protein-rich medium. However, the current state of *C*. *difficile* genetics requires that strains be isolated under nutrient-poor conditions. Because the growth of the *C*. *difficile selD* deletion may behave similarly to the TargeTron insertion, we sought to develop a mutagenesis technique that permits the isolation of mutants under nutrient-rich conditions. The CRISPR-Cas9 system was an attractive target. To design a Cas9-producing plasmid (Fig. 3), we placed a wild-type *cas9* gene, which was codon-optimized for expression in *C*. *difficile*, under the conditional expression of the tetracycline-inducible *tetR* promoter^18^. Also, based on the finding that non-homologous end-joining in *C*. *cellulolyticum* is inefficient^29^, we engineered *cas9*_*D10A*_ to determine if the native double-stranded break repair system in *C*. *difficile* is also inefficient; the Cas9_D10A_ protein functions as a ‘nickase’ and does not introduce double-stranded breaks into the targeted DNA^30^.

The expression of the single guide RNA (sgRNA) that directs Cas9 to the intended target site was placed under the control of the native glutamate dehydrogenase (*gdh*) promoter^31,32^. This promoter is constitutively expressed and allows for sufficient levels of the sgRNA to be present within the cell for Cas9 to act upon. We engineered the sgRNA to be produced as a single RNA molecule by fusing the crRNA and tracrRNA, as previously described^33^. Potential crRNA target sites were determined using an algorithm provided by the CRISPRscan.org website^34^ and sites were chosen within the first 200 bp of the gene.

The final portion of the *C*. *difficile* CRISPR-Cas9 plasmid is the donor region which is necessary to insert a desired mutation. This 2-kb region (one kb upstream of the targeted DNA and one kb downstream of the targeted DNA) surrounds the region to be deleted. The function of this donor region is to provide a template for the native DNA repair system to correct the Cas9-mediated double-stranded DNA (dsDNA) or single-stranded DNA (ssDNA) break.

### Isolation of a CRISPR-Cas9 mediated *pyrE* mutation

**Figure 4:**
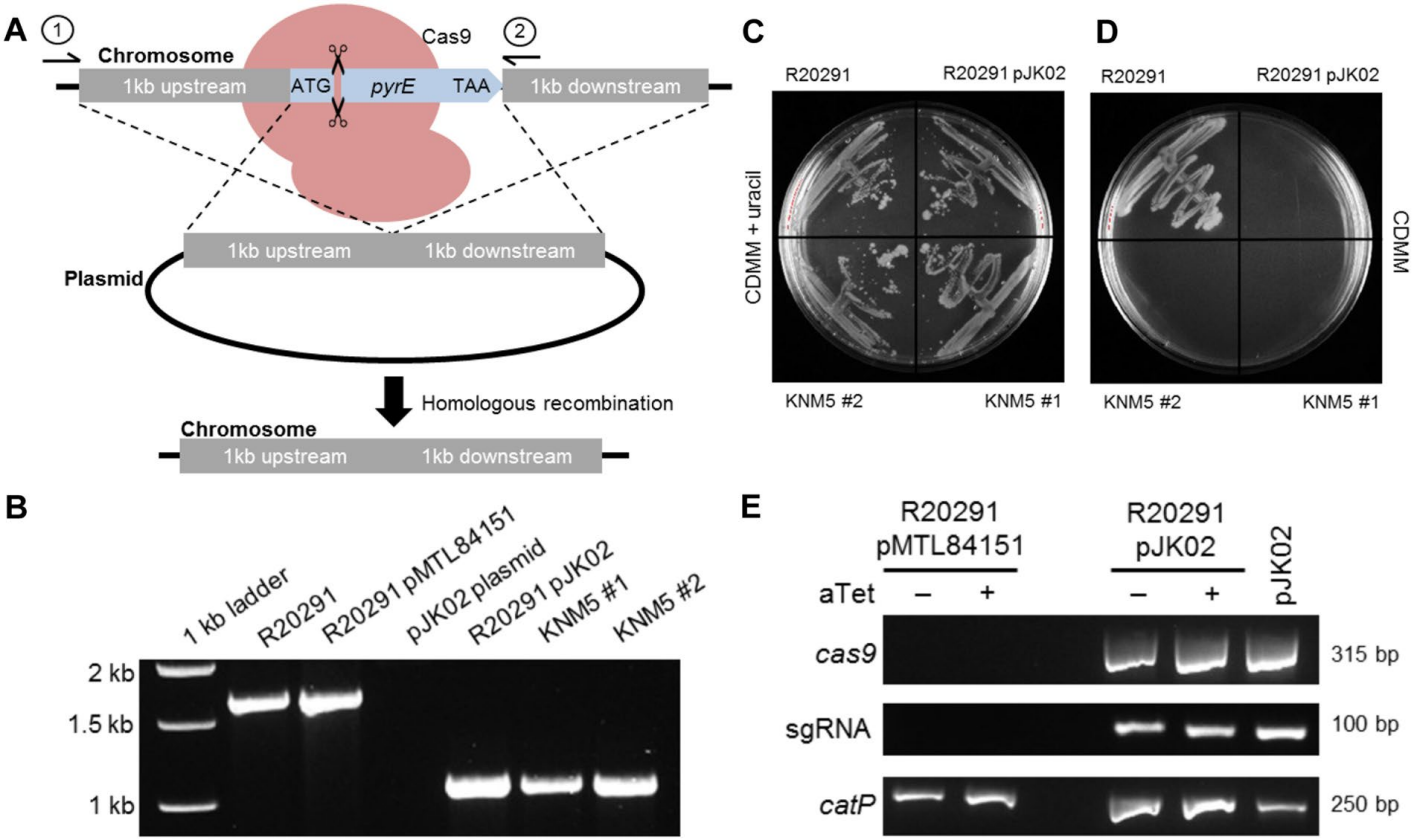
Isolating Cas9-mediated *C*. *difficile pyrE* mutants. (**A**) A deletion of the chromosomally-encoded *pyrE* gene was made by homologous recombination from a donor region located on the CRISPR-Cas9 plasmid during repair of a Cas9-mediated double-stranded DNA break. The location of the crRNA target region in *pyrE* is indicated by the cut DNA. Amplification of wildtype *pyrE* using primers 1 and 2 results in a 1.59 kb band on an agarose gel. (**B**) DNA was isolated from potential mutants. The region surrounding the *pyrE* gene was amplified from the chromosome, and the resulting DNA was separated on an agarose gel. The full, uncropped gel image can be found in the supplemental data. A clean deletion of *pyrE* is indicated by a faster-migrating DNA band. (**C**–**D**) *C*. *difficile* R20291, *C*. *difficile* R20291 pJK02, *C*. *difficile* KNM5 isolate 1 and *C*. *difficile* KNM5 isolate 2 were streaked onto either (**C**) CDMM supplemented with uracil or (**D**) CDMM alone, and incubated anaerobically for 4 days. (**E**) RT-PCR showing a comparison of *C*. *difficile* R20291 pMTL8151 and *C*. *difficile* R20291 pJK02 induced without aTet and those induced in the presence of aTet to turn on the expression of *cas9*. Also tested was the sgRNA and *catP*, as a positive control. The white dividing bar between R20291 pMTL84151 and R20291 pJK02 samples indicates an empty lane between samples. The white dividing bar between amplified genes indicates different gels. The full, uncropped gels can be found in the supplemental data.

To test the efficiency of the system in *C*. *difficile*, the first gene targeted for deletion was *pyrE*. The *pyrE* mutant strain is a uracil auxotroph and resistant to FOA-mediated toxicity. Therefore, mutations can easily be selected by incorporating FOA into growth medium. To engineer the deletion, we cloned 1-kb upstream of the *pyrE* start codon and 1-kb downstream of the stop codon in the mutagenesis plasmid (Fig. 4A). Next, a site near the 5′ end of the *pyrE* sequence was chosen for the crRNA. The resulting plasmid, pJK02, was introduced into *C*. *difficile* R20291 by conjugation from *E*. *coli*. The *tetR* promoter system was induced by aTet and mutants were isolated by selecting for those that were resistant to FOA. Colonies containing the mutation were confirmed by PCR amplification of the *pyrE* gene and surrounding DNA (Fig. 4B). A mutation in *pyrE* results in a 585-bp deletion within the 1.59 kb *pyrE* coding sequence (Fig. 4A and B). Subsequently, the phenotype of the *pyrE* mutant strain, *C*. *difficile* KNM5, was confirmed by plating on defined medium or medium supplemented with uracil (Fig. 4C and D). Only the wild-type strain was able to grow on medium without uracil supplementation. Surprisingly, we observed a *pyrE* deletion in the uninduced R20291 (pJK02) strain, which was to be used here as a control (Fig. 4B). This strain was also a uracil auxotroph (Fig. 4C and D). Based on results from a previous study^35^, we hypothesized that the *tetR* promoter has leaky expression and could lead to a small amount of transcription of *cas9* in the absence of aTet. To confirm this, we extracted RNA from an uninduced culture and amplified a portion of *cas9* (Fig. 4E). As expected, in the absence of induction, we observed *cas9* transcript without amplification of contaminating DNA (Fig. S1). These results suggest that the *tetR* promoter is not tightly regulated and uncontrolled expression of *cas9* led to a deletion of *pyrE* in the uninduced strain.

Incorporation of FOA in the growth medium permits the selection against *pyrE*-containing strains, but not all mutations lend themselves to a selection process. Thus, we determined the efficiency of the CRISPR-Cas9-mediated *pyrE* mutations. *C*. *difficile* R20291 containing the CRISPR-Cas9 plasmid (pJK02) was induced and the resulting cells were plated on defined medium alone or medium supplemented with FOA. The number of colonies were enumerated and the efficiency of mutagenesis was calculated (Table 1). Surprisingly, wild-type Cas9 yielded a mutation frequency of approximately 50%. Moreover, this mutation frequency was dependent on the ability of Cas9 to introduce a dsDNA break at the target site. That is, the Cas9_D10A_ protein yielded a much lower mutation frequency (~2 × 10^−4^, or 1 mutation in every 5,000 cells). This mutation frequency is above the empty vector control (~4 × 10^−7^), suggesting that *C*. *difficile* R20291 can use homology-directed repair to replace ssDNA breaks, but not efficiently. Importantly, the high mutation frequency of the wild-type Cas9 plasmid was not merely due to homologous recombination of the plasmid with the genome. If this were the case, the *cas9*_*D10A*_ plasmid should have yielded a similar frequency. These results suggest that the wild-type *cas9*-containing plasmid is best suited for introducing mutations into the *C*. *difficile* genome.

**Table 1:** Efficiencies for CRISPR-Cas9-mediated *pyrE* mutations in *C*. *difficile* R20291.

Strain	Total cell count (CFU/mL)	Mutant cell count (CFU/mL)	Calculated efficiencies	Average
R20291 pMTL84151 (empty vector)	3.5 × 10^8^	1.0 × 10^2^	2.86 × 10^−7^	4.42 × 10^−7^ ± 2.46 × 10^−7^
	1.9 × 10^8^	1.5 × 10^2^	7.89 × 10^−7^	
	2.0 × 10^8^	5.0 × 10^1^	2.50 × 10^−7^	
R20291 pKM93 (*cas9*_D10A_)	1.9 × 10^7^	3.0 × 10^3^	1.58 × 10^−4^	2.04 × 10^−4^ ± 5.27 × 10^−5^
	9.0 × 10^7^	2.5 × 10^4^	2.78 × 10^−4^	
	8.5 × 10^7^	1.5 × 10^4^	1.76 × 10^−4^	
R20291 pJK02 (WT *cas9*)	8.1 × 10^6^	4.0 × 10^6^	0.494	0.495 ± 0.049
	8.1 × 10^6^	4.5 × 10^6^	0.556	
	9.9 × 10^6^	4.3 × 10^6^	0.434	

### No off-target effects in *C*. *difficile* KNM5

**Figure 5:**
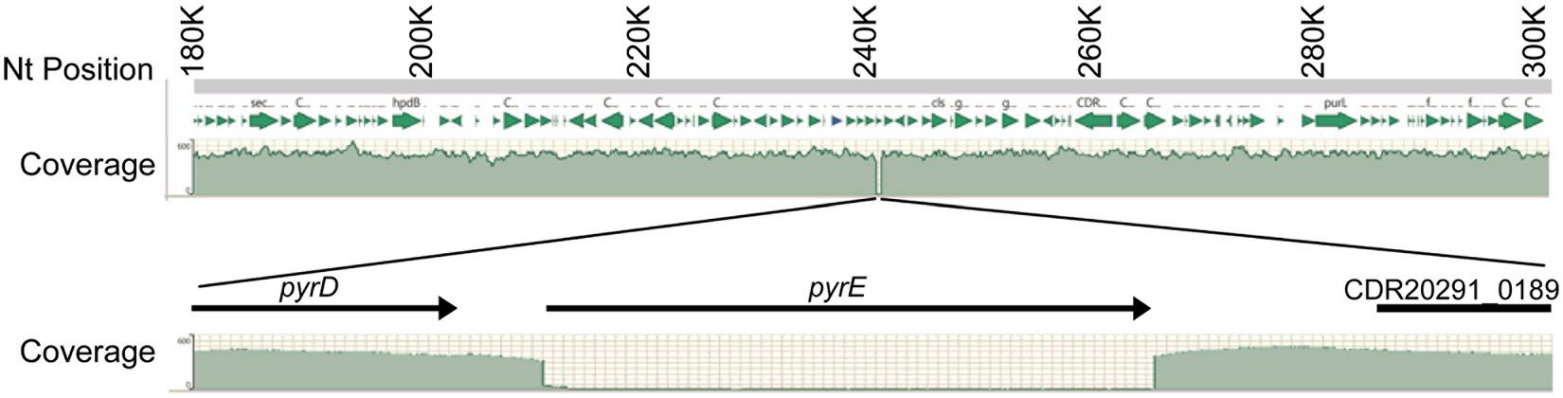
Monitoring off-target effects of the CRISPR-Cas9 system. Coverage of the sequencing reads from *C*. *difficile* KNM5 (Δ*pyrE*) in relation to their position on the *C*. *difficile* R20291 chromosome. The gap in the coverage is expanded along with annotations for the region.

To understand if the generated *pyrE* deletion mutants had other mutations in the genome, Illumina genome re-sequencing was performed for *C*. *difficile* KNM5 (Δ*pyrE*) and *C*. *difficile* R20291 (pMTL84151) (empty vector) after induction for the CRISPR-Cas9 system to account for any mutations that may have occurred due to the induction procedure (though *C*. *difficile* R20291 (pMTL84151) does not contain a region that could be used for homology directed repair, FOA-resistant colonies were observed on FOA-containing medium). As shown in Fig. 5, >300 reads were observed for every position across the genome (only the region surrounding *pyrE* is shown). However, at the *pyrE* gene, the reads drop drastically to ~20–30 reads, dropping further to undetectable levels until the end of the stop codon. The reads increase again after the stop codon; there were no other mutations in the KNM5 strains. Interestingly, the *C*. *difficile* R20291 (pMTL84151) FOA-resistant strain had a single-nucleotide polymorphism (SNP) in *pyrE* which resulted in a premature stop codon. These results suggest that there were no off-target effects generated by the CRISPR-Cas9 system and that it could be applied to other genes.

### Deletion of *selD* using the CRISPR-Cas9 system

Because the TargeTron mutation results in the insertion of the group II intron into the *selD* gene, there are likely polar effects on the downstream *selA* and *selB* sequences. To generate a deletion in *selD*, we targeted the sgRNA to the *selD* gene and cloned the upstream and downstream regions for use in homology-directed repair. This plasmid was introduced into *C*. *difficile* R20291, the resulting strain was induced and cells were plated directly on BHIS medium (rich medium). Colonies that grew were tested directly for the desired mutation by PCR. Though the efficiency of mutation was lower compared to *pyrE*, we observed a frequency of ~1 *selD* deletion in every 5 colonies tested (~20% mutation frequency) (Table 2).

**Table 2:** Efficiencies for CRISPR-Cas9-mediated *selD* deletion in *C*. *difficile* R20291.

Strain	Total colonies tested	PCR positive mutants	Calculated efficiencies	Average
R20291 pJS194 (*selD* target)	16	2	0.125	0.194 ± 0.161
	24	1	0.042	
	24	10	0.417	

**Figure 6:**
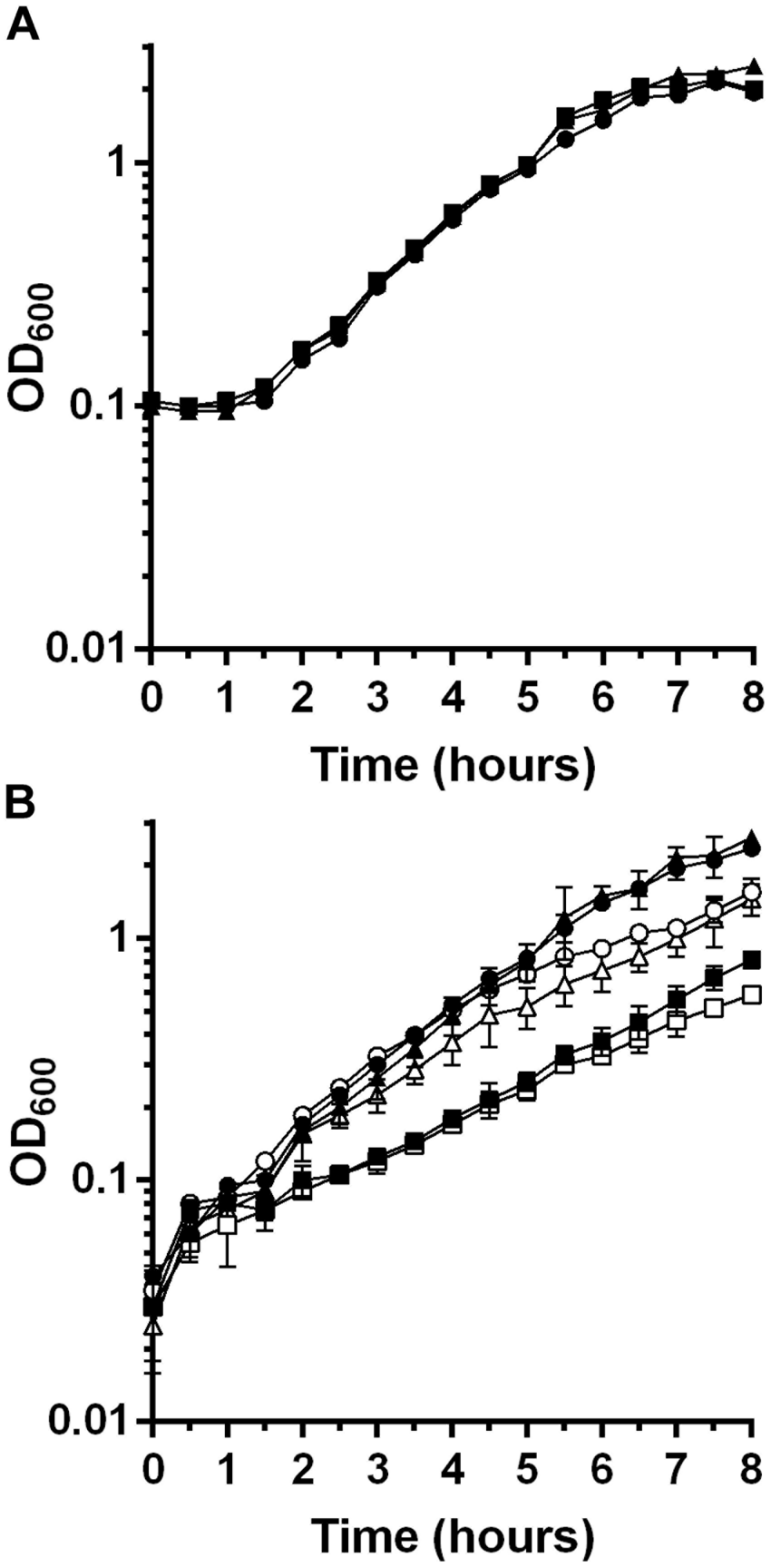
*C*. *difficile* Δ*selD* has a moderate growth defect compared to the WT strain. (**A**) *C*. *difficile* R20291 pJS116 (empty vector) (●), *C*. *difficile* KNM6 (Δ*selD*) pJS116 (empty vector) (■) and *C*. *difficile* KNM6 pKM142 (*selD* complement) (▲) were grown in BHIS medium and growth was monitored over time. (**B**) *C*. *difficile* R20291 pJS116, KNM6 pJS116 and KNM6 pKM142 were grown in TYG (closed shapes, ●) or TY (open shapes, ○) medium and growth was monitored over time. Data points represent the average from two independent experiments and error bars represent the standard deviation of the mean.

We then tested the growth phenotype of *C*. *difficile* KNM6 (Δ*selD*) and compared it to the growth observed for the wild-type R20291 strain. In the same experiment, we tested whether there were no polar effects on downstream genes in the operon, *selA* and *selB*, from this clean deletion of *selD*. To do this, we introduced *selD* with its native promoter on a multi-copy plasmid, *C*. *difficile* KNM6 pKM142. We included empty vector, pJS116, in wild-type R20291 as well as the mutant KNM6 strains. When grown in rich, BHIS medium (Fig. 6A), no difference is seen between the three strains, R20291 pJS116, KNM6 pJS116, and KNM6 pKM142. When grown in medium that may favor Stickland metabolism (TY or TYG medium), the wild-type strain with empty vector, R20291 pJS116, grew well while the mutant strain with empty vector, KNM6 pJS116, had a reduction in growth in comparison (Fig. 6B). When this strain was complemented by expressing *selD*, not the entire locus, KNM6 pKM142 grew to wild-type levels (Fig. 6B). This confirms there were no polar effects on the downstream genes, *selA* and *selB*, due to the deletion of *selD*.

Taken together, our results suggest that selenium incorporation into proteins is important for *C*. *difficile* growth and that CRISPR-Cas9 gene editing can be used to rapidly and efficiently introduce mutations with no polar effects into the *C*. *difficile* genome.

## Discussion

CRISPRs were originally discovered in *Escherichia coli* and later in archaea and other bacteria, including *C*. *difficile* ^36–38^. The development of utilizing CRISPRs and Cas proteins has led to functional gene editing tools that are widely used. For gene editing in bacteria, the components necessary for this system include: a Cas protein, a guide RNA, and a region of donor DNA to make the desired mutation^33,39^. A short sequence proximal to the target sequence, which helps the CRISPR-*cas9* system to distinguish between self and non-self-sequences, is called the protospacer adjacent motif (PAM) sequence^40^. *S*. *pyogenes* Cas9 recognizes the 5′–NGG–3′ PAM sequence^40,41^.

*C*. *difficile* encodes a native CRISPR-Cas system and belongs to the class I-B subtype^38^. The *S*. *pyogenes* Cas9, used in this study, belongs to the class II group. The classes are defined by their mechanisms and also the composition of Cas proteins^33^; thus, *C*. *difficile* has different Cas proteins than that of *S*. *pyogenes*. The CRISPR-Cas system of *C*. *difficile* is predicted to recognize a PAM sequence of 5′–CCW–3′, where “W” indicates either an adenine (A) or thymine (T)^38^. Due to the differences in PAM recognition sequences of these two CRISPR-Cas systems, we do not predict that the *C*. *difficile* CRISPR-Cas locus will interfere with this genetic tool.

We successfully developed the first application of a CRISPR-Cas9 system for genetic modification in *C*. *difficile*. Due to the problems inherent to each genetic system used in *C*. *difficile*, we wanted to create a plasmid containing the system which was simple and easy to modify for future use by others in the field. Towards this goal, the fragments to generate the homology region can be cloned in one step using Gibson Assembly; a gBlock containing the entirety of the sgRNA is also cloned into the plasmid using Gibson Assembly, each at unique restriction sites in the plasmid. Thus, a new mutagenesis plasmid can be generated in two consecutive cloning steps.

We used the *pyrE* gene as a starting point to optimize the system. By doing so, we were able to determine that *C*. *difficile* has a very poor ssDNA break repair system, as evident by the low efficiency of the Cas9_D10A_-mediated *pyrE* deletion. However, Cas9_D10A_ had a greater frequency of FOA-resistant colonies than did *C*. *difficile* R20291 pMTL84151 (empty vector), suggesting that *C*. *difficile* can use homology-directed repair to correct ssDNA breaks, but not at an efficiency which could allow for the isolation of mutants without selective pressure. Thus, the *cas9*_*D10A*_ allele is not a viable option for this CRISPR-based genetic system.

Moving forward with the wild-type Cas9, a concern was whether the mutations were due simply to homologous recombination between the chromosome and the donor DNA on the plasmid or required the repair of the CRISPR-Cas9-mediated dsDNA break. The efficiency of FOA-resistant cells (*pyrE* mutants) was greater for strains containing wild-type Cas9 than for strains that contained the *cas9*_*D10A*_ allele. Thus, without the aid of the wild-type Cas9 nuclease, *C*. *difficile* cannot introduce the desired mutation by homologous recombination with a high enough efficiency to allow for isolation of a mutant without a selection.

Selenophosphate synthetase is an enzyme that uses ATP, water, inorganic phosphate and hydrogen selenide to generate selenophosphate^26,42^. Selenophosphate is used as a donor to generate selenocysteine-charged tRNAs by attaching selenide to serine-charged tRNAs, leading to incorporation of selenocysteine into selenoproteins (*e*.*g*., PrdB or GrdA)^26,43^. Previously, a *prdB* mutant was shown to have a decreased growth rate compared to the parent strain^22^. We had hypothesized that, because SelD is required for generating the precursor to selenocysteine-charged tRNAs, the CRISPR *selD* mutant would have a greater effect on growth rate than a *prdB* mutation due to the global reduction in selenoproteins. Indeed both the TargeTron mutant and the CRISPR-generated *selD* deletion had reduced growth in protein-rich medium (medium where Stickland metabolism is important for growth). We also show there were no polar effects on the downstream *selA* and *selB* genes from the clean deletion of *selD*. In the future, this mutant will help in studying the global effect of the disruption of selenium incorporation into proteins in *C*. *difficile*, not just growth and metabolism.

The mutation efficiencies for *pyrE* and *selD* were similar and both were well within testable limits. The crRNA chosen for *pyrE* began at the 36^th^ nucleotide of the 585-bp *pyrE* gene and had a score in CRISPRscan.org of 30, which is low compared to the highest score of 62 for a crRNA for this gene. The crRNA chosen for *selD* had a score in CRISPRscan.org of 67, the highest listed, and started at the 183^rd^ nucleotide of the 951-bp *selD* gene. From these values for the respective genes, there appears to be no pattern for how efficient the CRISPR-Cas9 system in *C*. *difficile* can make the mutation. The rules for choosing the optimal crRNA that will yield the highest efficiency for generating a mutation in *C*. *difficile* is still under investigation.

In summary, we have developed a functional CRISPR-Cas9 system for use in *C*. *difficile*. Because other systems rely on the integration of segregationally-unstable plasmids into the genome, an event that can take several passages and the eventual regeneration of a chromosomal deletion in *pyrE*, this CRISPR-based plasmid has the potential to rapidly generate mutations within the *C*. *difficile* genome. With future adjustments to this system, a larger range of mutations, insertions and even point mutations, could possibly be made in *C*. *difficile* which has been difficult or even impossible in the past.

## Materials and Methods

### Bacterial strains and growth conditions

*C*. *difficile* strains (Table S1) were routinely grown in an anaerobic atmosphere (10% H_2_, 5% CO_2_, 85% N_2_) at 37 °C in brain heart infusion medium supplemented with 5 g/L yeast extract and 0.1% L-cysteine (BHIS), as described previously^7,44–46^ or TY medium (3% tryptone, 2% yeast extract)^47^. For conjugation experiments, cells were plated on BHI agar medium, as described previously for *E*. *coli*-based conjugations^48^, or on TY medium for *Bacillus subtilis*-based conjugations. For selenium incorporation (see below), strains were grown in TY (1% tryptone, 0.5% yeast extract)^24^. Where indicated, growth was supplemented with taurocholate (TA; 0.1% w/v), thiamphenicol (10 µg/mL), kanamycin (50 µg/mL), D-cycloserine (250 µg/mL), erythromycin (5 µg/mL) and/or glucose (1% w/v) as needed. Induction of the CRISPR-Cas9 system was performed in TY medium^47^ supplemented with thiamphenicol (10 µg/mL) and anhydrous tetracycline (aTet; 100 ng/mL). A defined minimal medium for *C*. *difficile* growth (CDMM), described previously^12,49^, was used for selection of *pyrE* mutants by supplementation with 5-fluoroorotic acid (FOA; 2 mg/mL) and uracil (5 µg/mL). *E*. *coli* strains (Table S1) were routinely grown at 37 °C in LB medium. Strains were supplemented with chloramphenicol (20 µg/mL), kanamycin (50 µg/mL), and/or ampicillin (100 µg/mL) as needed. *B*. *subtilis* BS49 was routinely grown at 37 °C in BHIS broth or on LB agar plates. Strains were supplemented with chloramphenicol (2.5 µg/mL) and/or tetracycline (5 µg/mL).

### Plasmid construction and molecular biology

The JIR8094 *selD* TargeTron insertion was created in several steps. First, plasmid pBL38 was constructed by retargeting of the group II intron from pCE240^50^, using the primers oLB70, oLB71, oLB72 and EBS-Universal, as outlined in the TargeTron user manual (Sigma-Aldrich), followed by initial cloning of the retargeted fragment in pCE240 digested with *BsrG*I and *Hind*III. The retargeted group II intron from pBL38 was then extracted by digestion with *Sfo*I and *Sph*I and cloned between the *Sph*I and *SnaB*I sites of pMC123^51^, resulting in pBL54. Conjugation experiments between *C*. *difficile* and *E*. *coli* were carried out as described previously^52^. *C*. *difficile* transconjugants were selected on BHIS plates supplemented with D-cycloserine, kanamycin, and thiamphenicol and potential TargeTron mutants were identified by plating on erythromycin. Erythromycin-resistant colonies were screened for the insertion of the intron into *C*. *difficile selD* by PCR using primers specific for full-length *C*. *difficile selD* (oLB76 and oLB77). A positive clone, strain LB-CD7, was identified. To identify if the TargeTron system integrated at sites other than the *selD* gene, we sequenced the *C*. *difficile* LB-CD7 strain and the parent JIR8094 strain. No additional TargeTron insertions were found except the one in *selD*.

To construct the CRISPR-*cas9* plasmid, the constitutively-expressed *cwp2* promoter was chosen to drive expression of a sgRNA targeted to *spoVAC*. Oligonucleotides were designed and the fragments generated were stitched together by PCR SOEing with Phusion DNA polymerase (due to the nature of the A: T-rich sequence, the entire fragment could not be synthesized directly and thus had to be stitched together by PCR) (all oligonucleotide sequences can be found in Table S2). The first round of amplification was done with primer sets gRNA_1_for and gRNA_2_rev, gRNA_3_for and gRNA_4_rev, and gRNA_5_for and gRNA_6_rev. The resulting fragments, gRNA_1_2 and gRNA_3_4, were used along with primers gRNA_1_for and gRNA _4_rev in a PCR to yield fragment gRNA_1_4. Fragment gRNA_5_6 was expanded in a PCR using primers gRNA_5_for and gRNA_7_rev to yield fragment gRNA_5_7. The complete sgRNA was made by PCR sowing of fragments gRNA_1_4 and gRNA_5_7 using primers 5′gRNA and 3′gRNA. This DNA fragment was introduced into pJS116 (all plasmid descriptions can be found in Table S1) at the *Pme*I restriction site using Gibson Assembly^53^ and transformed into *E*. *coli* DH5α to generate pKM22. The *tetR* repressor gene along with the P_tet_ promoter for conditional expression of *cas9* was amplified by PCR from pRPF215^18^ (Table S1) using primers 5′MTL_tetRprom and 3′tetR_Cas9. *cas9* from *Streptococcus pyogenes* was codon optimized for expression in *C*. *difficile* by Thermo Fisher (Thermo Fisher Scientific, Waltham, MA). Codon-optimized *cas9* was amplified using primers 5′tetR_CO_cas9 and 3′MTL_CO_cas9 from pMK-RQ-Bs-cas9. To introduce a D10A mutation into *cas9*, we used primer set 5′tetR_CO_cas9_D10A and 3′MTL_CO_cas9 for PCR amplification. The P_tet_ promoter and the wild-type *cas9* and *cas9*_*D10A*_ alleles were introduced into pKM22 at the *Hind*III restriction site using Gibson Assembly to generate pKM46 and pKM48, respectively.

To facilitate future crRNA changes and increase the expression of the sgRNA, the stronger, constitutively-expressed *gdh* promoter was used to replace the *cwp2* promoter. Also, *Kpn*I and *Mlu*I restriction sites were added at the 5′ and 3′ ends of the sgRNA, respectively. 5′gdh and 3′gdh_gRNA were used to amplify the *gdh* promoter from *C*. *difficile* R20291. The sgRNA was amplified from pKM22 using primers 5′gRNA_gdh and 3′gRNA 2. The resulting two fragments were both inserted into the *Pme*I and *BsrG*I sites using Gibson Assembly and transformed into *E*. *coli* DH5α to generate pKM54 and pKM55.

In order to easily select for mutants, we designed a plasmid to target the *pyrE* gene. The donor region for homology directed repair was made such that 1-kb upstream and 1-kb downstream were stitched together to generate a clean deletion of *pyrE*. 1-kb upstream and 1-kb downstream of *pyrE* were separately amplified by PCR from *C*. *difficile* R20291 using primers 5′pyrE_UP and 3′pyrE_UP and 5′pyrE_DOWN and 3′pyrE_DOWN, respectively. The two resulting fragments were inserted into the *Not*I and *Xho*I restriction sites in pKM54 and pKM55 background by Gibson Assembly and transformed into *E*. *coli* DH5α to generate pKM64 and pKM65, respectively. A gBlock (Integrated DNA Technologies, Coralville, IA), pyrE_gRNA_gBlock, was designed which contained the sgRNA DNA sequence between, and including, the *Kpn*I restriction site and the *Mlu*I site. This DNA fragment was introduced between the *Kpn*I and *Mlu*I restriction sites of pKM64 and pKM65 to generate pKM71 and pKM72, respectively. To introduce the plasmids into *C*. *difficile* by conjugation with *E*. *coli*, the *B*. *subtilis Tn*916 *oriT* was replaced with the *E*. *coli traJ* gene. *traJ* was amplified from pMTL84151 by PCR using primers 5′traJ and 3′traJ. The resulting fragment was introduced into pKM71 and pKM72 using the *Apa*I restriction site by Gibson Assembly and transformed into *E*. *coli* DH5α to generate pJK02 (accession number MF782679) and pKM93 respectively.

The subsequent CRISPR-Cas9 plasmid targeting *selD* was made using pJK02. The homology regions for *selD* targeting were amplified by primer sets 5′MTL_selD_UP and 3′MTL_selD_UP and 5′MTL_selD_DN and 3′MTL_selD_DN. The resulting fragments were cloned by Gibson assembly into pJK02 at the *Not*I and *Xho*I restriction sites and transformed into *E*. *coli* DH5α resulting in pJS170. The gBlock for *selD* targeting sgRNA, CRISPR_selD_183, was introduced by ligation into the *KpnI* and *MluI* sites and transformed into *E*. *coli* DH5α resulting in pJS187. The *selD* targeting plasmid was modified by replacing *traJ* with oriT *tn*916 for *B*. *subtilis* conjugation by amplification from pJS116 using primers 5′Tn916ori and 3′Tn916ori. The resulting fragment was introduced into pJS187 by Gibson assembly at the *Apa*I site and transformed into *E*. *coli* DH5α resulting in pJS194.

To make a plasmid which would complement the *selD* mutation, *selD* along with 500 bp upstream to include the native promoter was amplified by primer sets 5′selD_comp and 3′selD_comp. The resulting fragment was cloned by Gibson assembly into pJS116 at the *Not*I and *Xho*I restriction sites and transformed into E. coli DH5α resulting in pKM142. The sequences of all plasmids were verified by DNA sequencing.

### Conjugation for CRISPR-Cas9 and complementation plasmid insertion

All complete CRISPR-Cas9 and complement plasmids were transformed into either *E*. *coli* HB101 pRK24 or *B*. *subtilis* BS49 to be used as donor for conjugation with *C*. *difficile*. For *E*. *coli* conjugations, the strains were grown overnight at 37 °C in LB supplemented with ampicillin and chloramphenicol. *C*. *difficile* R20291 was grown anaerobically in TY medium overnight. Five hundred microliters of *C*. *difficile* overnight culture/mating was heated to either 52 °C for 5 minutes for an 8 hour conjugation or 50 °C for 15 minutes for a 24 hour conjugation, as described previously^48^. *C*. *difficile* cultures were removed from the heat block and let cool to 37 °C for 2 minutes. Meanwhile, 1 mL of *E*. *coli* HB101 pRK24 containing the CRISPR-Cas9 plasmid cultures were pelleted at 4,000 × g for 2 minutes and the supernatant was removed. The *E*. *coli* pellets were transferred to the anaerobic chamber and gently suspended in the heat shocked *C*. *difficile* sample. The resulting mix was plated onto pre-reduced BHI agar plates by spotting ten, 20 µL drops of culture. After either 8 or 24 hours, the growth was harvested by collecting in 1 mL pre-reduced TY broth. One hundred microliters of the resuspended growth was plated onto multiple BHIS agar plates supplemented with thiamphenicol, kanamycin, and D-cycloserine. Growth was monitored for 2 to 3 days. Individual colonies were restreaked for isolation and tested for insertion of plasmid by PCR amplification of the *catP* gene with primers 5′ catP 3 and 3′ catP 2.

For *B*. *subtilis* conjugation, *C*. *difficile* R20291 was grown anaerobically in BHIS broth overnight. The *C*. *difficile* overnight culture was diluted in fresh pre-reduced BHIS broth and grown anaerobically for 4 hours. Meanwhile, *B*. *subtilis* BS49 was grown aerobically at 37 °C in BHIS broth supplemented with tetracycline and chloramphenicol for 4 hours. One hundred microliters of each culture was plated on TY agar medium. After 24 hours, the growth was harvested by suspending in 2 mL pre-reduced BHIS broth. One hundred microliters of the resuspended growth was spread onto several BHIS agar plates supplemented with thiamphenicol, kanamycin, and D-cycloserine. *C*. *difficile* transconjugants were screened for the presence of Tn*916* using tetracycline resistance, as described previously. Thiamphenicol-resistant, tetracycline-sensitive transconjugants were selected and used for further experiments.

### Radiolabeling studies with ^75^Selenium

Selenium is taken up with high affinity and specifically incorporated into macromolecules through exposure of cells to ^75^Se in the form of selenite^25^. For these studies, a 1:100 dilution of overnight cultures were added to 10 mL TY medium supplemented with approximately 10 µCi ^75^Se in the form of selenite (corresponding to 50 nM cold). After 24 hours growth, nine milliliter cultures were grown overnight in 12 × 75 mm capped culture tubes in an atmosphere of 95% nitrogen and 5% hydrogen. Cells were harvested by centrifugation (5,000 × g for 5 minutes) and resuspended in a small amount (typically 0.2 mL) of lysis buffer (50 mM Tris, pH 8.0, 0.1 mM benzamidine, 0.5 mM EDTA). Cells were lysed by sonication (model 100 Fisher Scientific) for short 10 second bursts until lysis was seen. The crude cell lysates were further clarified by centrifugation (12,500 × g) for 10 minutes at 4 °C. Protein concentrations were determined by Bradford assay^54^ using albumin to generate a standard curve. Radiolabeled selenoproteins were separated by SDS-PAGE (15% resolving gel) and, after the gels were dried, selenoproteins were identified by phosphorimager analysis (Molecular Dynamics phosphorimager).

### Induction of the CRISPR-Cas9 system and isolating mutants

*C*. *difficile* R20291 strains containing the *pyrE*-targeting plasmids were grown overnight in TY medium supplemented with thiamphenicol. In the morning, 250 µL of an overnight culture was diluted into 4.75 mL of fresh TY medium supplemented with thiamphenicol and aTet (100 ng/mL) and grown for 6 hours. Subsequently, cultures serially diluted and spread on CDMM medium supplemented with FOA and uracil. Colonies were isolated; DNA was extracted and tested for the desired mutation by PCR amplification of the target gene, 5′pyrE 2 and 3′pyrE 2 (Table S2). Once an isolate was confirmed, it was passaged ~3 times in BHIS liquid medium in order to lose the CRISPR-Cas9 plasmid. After pick-and-patch on BHIS agar with and without thiamphenicol, loss of plasmid was confirmed by PCR amplification of the *catP* gene using primer set 5′ catP 3 and 3′ catP 2. *C*. *difficile* R20291 strains containing the *selD* targeting plasmid was induced for 24 hours. Then ~10 µL of culture was spread onto BHIS medium. Colonies were tested as described above using primer sets 5′selD and 3′selD. Confirmed isolates were passaged on BHIS agar once in order to lose the CRISPR-Cas9 plasmid due to the slow growth of the mutant. Loss of plasmid was confirmed by PCR amplification of the *catP* gene using primer set 5′ catP 3 and 3′ catP 2.

### RT-PCR

RNA was extracted from wild-type *C*. *difficile* R20291 pMTL84151 (empty vector) and *C*. *difficile* R20291 pJK02 induced for 30 minutes with or without aTet using a FastRNA Blue Kit (MP Biomedical). DNA contamination was eliminated by using a TURBO DNA-*free* Kit (Thermo Scientific) according to the standard protocol. cDNA was made using the SuperScript III First-Strand Synthesis System (Thermo Scientific) according to the protocol, including controls for each sample without the presence of reverse transcriptase. To determine if *cas9* and the gRNA were being transcribed, the 5′ end of *cas9*, sgRNA, and *catP* were amplified from isolated cDNA in a PCR using primers sets 5′COcas9_RT and 3′COcas9_RT, 5′gRNA_RT and 3′gRNA_RT, and 5′catP_RT and 3′catP_RT and Taq DNA polymerase.

### Illumina sequencing

High-quality, high-molecular weight genomic DNA from *C*. *difficile* R20291 (WT), aTet-induced *C*. *difficile* R20291 pMTL84151 (empty vector), two isolates of *C*. *difficile* KNM5, *C*. *difficile* LB-CD7, *C*. *difficile* KNM6 pJS116, and *C*. *difficile* KNM6 pKM142 was extracted as described previously^52,55^. The genomic DNA was submitted to Tufts University School of Medicine Genomics Core facility for Paired-End 50 Illumina re-sequencing as described previously^7^. Alignment and analysis of the sequences was performed using DNASTAR Lasergene program MegAlign Pro 14.

### Determining mutation efficiencies

*C*. *difficile* R20291 strains containing the *pyrE* targeting CRISPR-Cas9 plasmids were induced as described above. Induced cultures were serially diluted and 100 µL was spread on CDMM supplemented with uracil and CDMM supplemented with FOA and uracil. After 4 days, colony forming units (CFUs) were counted for each dilution on each media and the total CFU/mL of the mutants (those on CDMM-FOA and uracil) and the total cell count (CDMM-uracil) were calculated.

*C*. *difficile* R20291 strains containing the *selD* targeting CRISPR-Cas9 plasmid was induced as described above. A loop containing ~10 µL of culture was spread onto rich BHIS agar medium. Individual colonies were isolated; DNA was extracted, and tested for the desired mutation by PCR amplification of the target genes using primers 5′selD and 3′selD (Table S2). These oligonucleotides only amplify DNA from the chromosome regardless of whether the CRISPR-Cas9 plasmid is present or not.

### Statistical Analysis

Data points represent the mean from two or three independent experiments and, where indicated, error bars represent one standard deviation from the mean.

### Availability of materials and data

The datasets generated during and/or analyzed during the current study are available in the NCBI SRA repository. The first accession number SRP115702 includes the following sequences and accession numbers: R20291 (SRX3104072), R20291 foaR (SRX3104073), and two KNM5 isolates (SRX3104071 and SRX3104074). The second accession number SRP119051 includes the following sequences and accession numbers: LB-CD7 (SRX3236353), KNM6 pJS116 (SRX3236354), and KNM6 pKM142 (SRX3236352). Finally, the pJK02 plasmid generated in this study is freely available to the scientific community.

## Electronic supplementary material

Supplemental Information

## References

[1] Oren A, Garrity GM (2016). Notification that new names of prokaryotes, new combinations, and new taxonomic opinions have appeared in volume 66, part 9, of the IJSEM. Int J Syst Evol Microbiol.

[2] Lawson PA, Citron DM, Tyrrell KL, Finegold SM (2016). Reclassification of Clostridium difficile as Clostridioides difficile (Hall and O’Toole 1935) Prevot 1938. Anaerobe.

[3] Rupnik M, Wilcox MH, Gerding DN (2009). *Clostridium difficile* infection: new developments in epidemiology and pathogenesis. Nature reviews. Microbiology.

[4] Smits WK, Lyras D, Lacy DB, Wilcox MH, Kuijper EJ (2016). *Clostridium difficile* infection. Nature Reviews Disease Primers.

[5] Theriot CM, Young VB (2015). Interactions Between the Gastrointestinal Microbiome and Clostridium difficile. Annual review of microbiology.

[6] Sorg JA, Sonenshein AL (2008). Bile salts and glycine as cogerminants for *Clostridium difficile* spores. J. Bacteriol..

[7] Francis MB, Allen CA, Shrestha R, Sorg JA (2013). Bile acid recognition by the *Clostridium difficile* germinant receptor, CspC, is important for establishing infection. PLoS pathogens.

[8] Paredes-Sabja D, Shen A, Sorg JA (2014). *Clostridium difficile* spore biology: sporulation, germination, and spore structural proteins. Trends in microbiology.

[9] Martin-Verstraete, I., Peltier, J. & Dupuy, B. The Regulatory Networks That Control Clostridium difficile Toxin Synthesis. *Toxins (Basel)* **8**, 10.3390/toxins8050153 (2016). 10.3390/toxins8050153 PMC4885068 27187475

[10] Poutanen SM, Simor AE (2004). *Clostridium difficile*-associated diarrhea in adults. CMAJ.

[11] Aktories, K., Schwan, C. & Jank, T. Clostridium difficile Toxin Biology. *Annual review of microbiology*, 10.1146/annurev-micro-090816-093458 (2017). 10.1146/annurev-micro-090816-093458 28657883

[12] Cartman ST, Kelly ML, Heeg D, Heap JT, Minton NP (2012). Precise manipulation of the *Clostridium difficile* chromosome reveals a lack of association between the tcdC genotype and toxin production. Applied and environmental microbiology.

[13] Ng YK (2013). Expanding the repertoire of gene tools for precise manipulation of the *Clostridium difficile* genome: allelic exchange using pyrE alleles. PloS one.

[14] Heap JT, Pennington OJ, Cartmant ST, Carter GP, Minton NP (2007). The ClosTron: A universal gene knock-out system for the genus *Clostridium*. J. Microbiol. Methods..

[15] Dineen SS, Villapakkam AC, Nordmant JT, Sonenshein AL (2007). Repression of *Clostridium difficile* toxin gene expression by CodY. Molecular Microbiology.

[16] O’Connor JR (2006). Construction and analysis of chromosomal *Clostridium difficile* mutants. Molecular Microbiology.

[17] Cartman, S. T. & Minton, N. P. A mariner-based transposon system for *in vivo* random mutagenesis of *Clostridium difficile*. *Appl*. *Environ*. *Microbiol*., AEM. 02525-02509, 10.1128/aem.02525-09 (2009). 10.1128/AEM.02525-09 PMC2820977 20023081

[18] Dembek M (2015). High-throughput analysis of gene essentiality and sporulation in Clostridium difficile. mBio.

[19] Heap JT (2010). The ClosTron: Mutagenesis in Clostridium refined and streamlined. J Microbiol Methods.

[20] Kelly ML (2016). Improving the reproducibility of the NAP1/B1/027 epidemic strain R20291 in the hamster model of infection. Anaerobe.

[21] Bakker D (2014). The HtrA-like protease CD3284 modulates virulence of Clostridium difficile. Infection and immunity.

[22] Bouillaut L, Self WT, Sonenshein AL (2013). Proline-dependent regulation of *Clostridium difficile* Stickland metabolism. J. Bacteriol..

[23] Bouillaut L, Dubois T, Sonenshein AL, Dupuy B (2015). Integration of metabolism and virulence in *Clostridium difficile*. Res Microbiol.

[24] Jackson S, Calos M, Myers A, Self WT (2006). Analysis of proline reduction in the nosocomial pathogen *Clostridium difficile*. Journal of Bacteriology.

[25] Self, W. T. Specific and nonspecific incorporation of selenium into macromolecules. *Comprehensive Natural Products Ii: Chemistry and Biology*, *Vol 5: Amino Acids*, *Peptides andProteins*, 121–148 (2010).

[26] Srivastava M, Mallard C, Barke T, Hancock LE, Self WT (2011). A selenium-dependent xanthine dehydrogenase triggers biofilm proliferation in *Enterococcus faecalis* through oxidant production. J Bacteriol.

[27] Power, D. A. & Zimbro, M. J. *Difco & BBL manual: manual of microbiological culture media*. 696 pages (Difco Laboratories, Division of Becton Dickinson and Co., 2003).

[28] Bouillaut L, Self WT, Sonenshein AL (2013). Proline-dependent regulation of Clostridium difficile Stickland metabolism. Journal of bacteriology.

[29] Xu T (2015). Efficient Genome Editing in *Clostridium cellulolyticum* via CRISPR-Cas9 Nickase. Applied and environmental microbiology.

[30] Shen B (2014). Efficient genome modification by CRISPR-Cas9 nickase with minimal off-target effects. Nat Methods.

[31] Mani N, Dupuy B (2001). Regulation of toxin synthesis in *Clostridium difficile* by an alternative RNA polymerase sigma factor. PNAS.

[32] Mani N, Dupuy B, Sonenshein AL (2006). Isolation of RNA polymerase from Clostridium difficile and characterization of glutamate dehydrogenase and rRNA gene promoters *in vitro* and *in vivo*. Journal of bacteriology.

[33] Doudna JA, Charpentier E (2014). Genome editing. The new frontier of genome engineering with CRISPR-Cas9. Science.

[34] Moreno-Mateos MA (2015). CRISPRscan: designing highly efficient sgRNAs for CRISPR-Cas9 targeting *in vivo*. Nature methods.

[35] Oliveira Paiva AM, Friggen AH, Hossein-Javaheri S, Smits WK (2016). The Signal Sequence of the Abundant Extracellular Metalloprotease PPEP-1 Can Be Used to Secrete Synthetic Reporter Proteins in Clostridium difficile. ACS Synth Biol.

[36] Ishino Y, Shinagawa H, Makino K, Amemura M, Nakata A (1987). Nucleotide sequence of the iap gene, responsible for alkaline phosphatase isozyme conversion in Escherichia coli, and identification of the gene product. Journal of bacteriology.

[37] Mojica FJ, Diez-Villasenor C, Soria E, Juez G (2000). Biological significance of a family of regularly spaced repeats in the genomes of Archaea, Bacteria and mitochondria. Molecular microbiology.

[38] Boudry P (2015). Function of the CRISPR-Cas System of the Human Pathogen *Clostridium difficile*. MBio.

[39] Sternberg SH, Doudna JA (2015). Expanding the Biologist’s Toolkit with CRISPR-Cas9. Molecular cell.

[40] Mojica FJ, Diez-Villasenor C, Garcia-Martinez J, Almendros C (2009). Short motif sequences determine the targets of the prokaryotic CRISPR defence system. Microbiology.

[41] Jinek M (2012). A programmable dual-RNA-guided DNA endonuclease in adaptive bacterial immunity. Science.

[42] Kim IY, Veres Z, Stadtman TC (1992). *Escherichia coli* mutant SELD enzymes. The cysteine 17 residue is essential for selenophosphate formation from ATP and selenide. J Biol Chem.

[43] Forchhammer K, Bock A (1991). Selenocysteine synthase from *Escherichia coli*. Analysis of the reaction sequence. J Biol Chem.

[44] Francis MB, Allen CA, Sorg JA (2015). Spore cortex hydrolysis precedes dipicolinic acid release during *Clostridium difficile* spore germination. J. Bacteriol..

[45] Allen CA, Babakhani F, Sears P, Nguyen L, Sorg JA (2013). Both fidaxomicin and vancomycin inhibit outgrowth of *Clostridium difficile* spores. Antimicrobial agents and chemotherapy.

[46] Sorg JA, Sonenshein AL (2009). Chenodeoxycholate is an inhibitor of *Clostridium difficile* spore germination. J. Bacteriol..

[47] Dupuy B, Sonenshein AL (1998). Regulated transcription of *Clostridium difficile* toxin genes. Molecular Microbiology.

[48] Kirk JA, Fagan RP (2016). Heat shock increases conjugation efficiency in Clostridium difficile. Anaerobe.

[49] Karlsson S, Burman LG, Akerlund T (1999). Suppression of toxin production in *Clostridium difficile* VPI 10463 by amino acids. Microbiology.

[50] Ho TD, Ellermeier CD (2011). PrsW is required for colonization, resistance to antimicrobial peptides, and expression of extracytoplasmic function sigma factors in *Clostridium difficile*. Infect Immun.

[51] McBride, S. M. & Sonenshein, A. L. Identification of a genetic locus responsible for antimicrobial peptide resistance in *Clostridium difficile*. *Infect*. *Immun*., IAI. 00731–00710, 10.1128/iai.00731-10 (2010). 10.1128/IAI.00731-10 PMC3019887 20974818

[52] Bouillaut, L., McBride, S. M. & Sorg, J. A. Genetic manipulation of Clostridium difficile. *Curr Protoc Microbio*l **Chapter** 9, Unit 9A 2, 10.1002/9780471729259.mc09a02s20 (2011). 10.1002/9780471729259.mc09a02s20 PMC3615975 21400677

[53] Gibson DG (2009). Enzymatic assembly of DNA molecules up to several hundred kilobases. Nature methods.

[54] Bradford MM (1976). A rapid and sensitive method for the quantitation of microgram quantities of protein utilizing the principle of protein-dye binding. Analytical biochemistry.

[55] Wren BW, Tabaqchali S (1987). Restriction endonuclease DNA analysis of Clostridium difficile. Journal of clinical microbiology.

