# Supplementary material for: Using CRISPR-Cas9-mediated genome editing to generate C. difficile mutants defective in selenoproteins synthesis
Source: Sci Rep. 2017 Nov 7;7:14672. doi: 10.1038/s41598-017-15236-5 (PMC5677094; doi:10.1038/s41598-017-15236-5)
Supplement: Supplementary file 1 — Supplemental Information [file 41598_2017_15236_MOESM1_ESM.pdf]

Using CRISPR-Cas9-mediated genome editing to generate *C. difficile* mutants defective in selenoproteins synthesis

Kathleen N. McAllister[1], Laurent Bouillaut[2,&], Jennifer N. Kahn[1], William T. Self[3] and Joseph A. Sorg[1]*

[1]Department of Biology, Texas A&M University, College Station, TX

[2]Department of Molecular Biology & Microbiology, Tufts University School of Medicine, Boston, MA

[3]Burnett School of Biomedical Sciences, University of Central Florida, Orlando, FL

[&]Present address: Matrivax R&D Corp. 650 Albany Street, Boston, MA

Running Title: CRISPR-Cas9 mutagenesis of *C. difficile*

*Corresponding Author

ph: 979-845-6299

email: jsorg@bio.tamu.edu

Supplemental Information

Figure S1: No DNA contamination in RNA samples of *C. difficile* R20291 vectors.

RT-PCR without reverse transcriptase showing the comparison of *C. difficile* R20291 pMTL8151 and *C. difficile* R20291 pJK02 induced without aTet and those induced in the presence of aTet to turn on the expression of *cas9*. Also tested was the sgRNA and *catP*, as positive controls. The full, uncropped gels corresponding to this data can be found below.

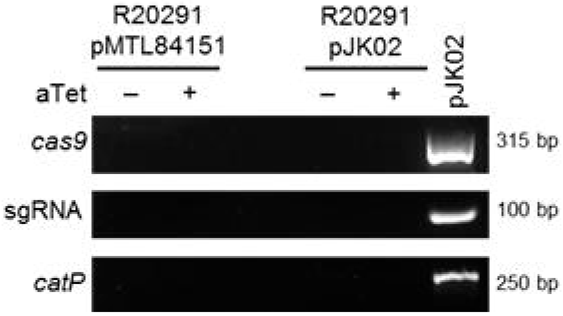

Table S1: Strains and plasmids used in this study.

Strain	Description/Phenotype	Source or Reference
E. coli DH5α	F^- endA1 glnV44 thi-1 recA1 relA1 gyrA96 deoR nupG Φ80d*lacZ*ΔM15 Δ(*lacZYA-argF*)U169, hsdR17(r_K^- m_K^+), λ–	[51]
E. coli HB101 pRK24	*lavYI galK2 xyl-6 mtl-I repsL20* carrying pRK24	B. Dupuy
B. subtilis BS49	Tn*916* donor strain, Tet^R	[52]
C. difficile JIR8094	*erm*-sensitive derivative of 630	[14]
C. difficile LB-CD7	*selD* TargeTron mutant	This Study

C. difficile LB-CD12	*grdA* TargeTron mutant	[19]
C. difficile LB-CD4	*prdB* TargeTron mutant	[19]
C. difficile LB-CD8	*prdR* TargeTron mutant	[19]
C. difficile R20291	Wild type, ribotype 027	[53]
C. difficile KNM5	*pyrE* targeted CRISPR-*cas9* mutant, uracil auxotroph	This study
C. difficile KNM6	*selD* targeted CRISPR-*cas9* mutant	This study
Plasmids		
pCE240	Derivative of pJIR750ai (Sigma-Aldrich)	[45]
pBL38	*selD* TargeTron in pCE240	This study
pBL54	pMC123-containing group II intron from pBL38	This study
pJK02	*traJ* in pKM71	This study
pJS116	*B. subtilis-C. difficile* shuttle vector (pCD6 ColE1 *Tn916 oriT* Cm^R)	[6]
pJS170	*selD* homology region in pJK02	This study
pJS187	*selD*-targeted sgRNA in pJS170	This study
pJS194	*tn916* oriT in pJS187	This study
pKM22	*spoVAC-* targeted sgRNA in pJS116	This study
pKM46	codon-optimized wildtype *cas9* in pKM22	This study
pKM48	codon-optimized $cas9_{D10A}$ in pKM22	This study
pKM54	*gdh* promoter with sgRNA in pKM46	This study
pKM55	*gdh* promoter with sgRNA in pKM48	This study
pKM64	*pyrE* homology region in pKM54	This study
pKM65	*pyrE* homology region in pKM55	This study
pKM71	*pyrE*-targeted sgRNA in pKM64	This study
pKM72	*pyrE*-targeted sgRNA in pKM65	This study

pKM93	*traJ* in pKM72	This study
pKM142	*selD* with 500 bp upstream in pJS116	This study
pMC123	*C. difficile* shuttle vector	[54]
pMK-RQ-Bs-cas9	codon-optimized *cas9* for *C. difficile* in pMK-RQ-Bs	Invitrogen, This study
pMTL84151	*E. coli*-*C. difficile* shuttle vector (pCD6 ColE1 *traJ* Cm^R)	[55]
pRPF215	tetracycline-inducible P_{tet} promoter	R. Fagan [16]

Table S2: Oligonucleotides used in this study.

Oligonucleotide	Sequence
gRNA_1_for	AAATACGGTGTTTTTTGTTACCCTAAGTTTCATAAAAATAAGAAGCCTGCAAATGCAGGCTTCTTATTTTTATGGTTTAAACCCGCATTATTAAA
gRNA_2_rev	CTTTTTCTTATATTTTATTTTTTTAATATTTATGTATACAAAAAAGTAACAAAATTGTAATTTTTTTTAATAATGCGGGTTTAAACCATAAAAAT
gRNA_3_for	AATATTAAAAAAATAAAATATAAGAAAAAGTTTATATCTTTTGGTTAATTATTACAATAAGTCTCATTTATTGAAATAATATCAAATATATATTA
gRNA_4_rev	ATTTCTAGCTCTAAAACAATAAAGAAAAAATCTATTTTTATTATTTTTTCCTTATTTACCAATTATAATATATATTTGATATTATTTCAATAAAT
gRNA_5_for	GATTTTTTCTTTATTGTTTTAGAGCTAGAAATAGCAAGTTAAAATAAGGCTAGTCCGTTATCAACTTGAAAAAGTGGCACCGAGTCGGTGCTTTT
gRNA_6_rev	TAAAAATAGTTGCAGAGCTTTGTACACTAGTCAGACATCATGCTGATCTAGATTTCTCCATAGAAAAAAAGCACCGACTCGGTGCCACTTTTTCA
gRNA_7_rev	ATTTTGGTCATGAGATTATCAAAAAGGAGTTTAATAAAAAATAAAAATAAGCTCTGCAACCATCTAAAAATAGTTGCAGAGCTTTGTACACTAGT
5'gRNA	AAATACGGTGTTTTTTGTTACCCTAAGTTTCATAAAAATAAGAAGCCTGCAAATGCAGGC
3'gRNA	TTGGTCATGAGATTATCAAAAAGGAGTTTAATAAAAAATAAAAATAAGCTCTGCAACCA
5'MTL_tetRprom	CCATGGAGATCTCGAGGCCTGCAGACATGCGCGATCGCAGACCCACTTTCACATTTAAG
3'tetR_Cas9	ATCTAAGCCTATTGAGTATTTCTTATCCATTTAATTAAAAAAACCTCCTAGTATTATTGAGC
5'tetR_CO_cas9	CTGAGCTCAATAATACTAGGAGGTTTTTTTAATTAAATGGATAAAAAATATAGTATAGGATTAGATATAGGAAC

3'MTL_CO_cas9	TCACGACGTTGTAAAACGACGGCCAGTGCCAAGCTTTTAATCACCACCTAATTGAGATAAATCTAT
5'tetR_CO_cas9_D10A	CTGAGCTCAATAATACTAGGAGGTTTTTTTAATTAAATGGATAAAAAATATAGTATAGGATTAGCTATAGGAACAAATAG
5'gdh	TGCAGGCTTCTTATTTTTATGGTTTAAACGGTTTTAGCTGGGATATCG
3'gdh_gRNA	AAAACAATAAAGAAAAAATCTATTTGGTACCTTTACAGTTTAATTATAGCACACTTTATT
5'gRNA_gdh	AGTGTGCTATAATTAAACTGTAAAGGTACCAAATAGATTTTTTCTTTATTGTTTTAG
3'gRNA 2	CATCTAAAAATAGTTGCAGAGCTTACGCGTCTAGTCAGACATCATGCTGA
5'pyrE_UP	TTATCAGGAAACAGCTATGACCGCGGCCGCGACGTGATTTTTAATGGGTA
3'pyrE_UP	TAAGTAACACTATAAATAATTAAGTTTTTAATTATTTTTCCTCCATGTTAATG
5'pyrE_DOWN	ATAACATTAACATGGAGGAAAAATAATTAAAAACTTAATTATTTATAGTGTTACTTAAAA
3'pyrE_DOWN	GTGGGTCTGCGATCGCGCATGTCTGCAGGCCTCGAGAAGCATTGATGTTCTTCCT
pyrE_gRNA_gBlock	GGTACCGAAAAGTGATGCATTGTTGGGTTTTAGAGCTAGAAATAGCAAGTTAAAATAAGGCTAGTCCGTTATCAACTTGAAAAAGTGGCACCGAGTCGGTGCTTTTTTTCTATGGAGAAATCTAGATCAGCATGATGTCTGACTAGACGCGT
5'traJ	GCGAGGAAGCGGAAGAGCGCCCAATACGCAGGGCCCCCTGCTTCGGGGTCA
3'traJ	AATTTATCTACAATTTTTTTATCCTGCAGGGGGCCCGATCGGTCTTGCCTTG
5'MTL_selD_UP	TTATCAGGAAACAGCTATGACCGCGGCCGCATTAATAAAAGTGATAAATTTCTTTTCAT
3'MTL_selD_UP	TCAAAACAATCACTCTTTCTCTATAATATTTTGAGATAAAACTGATGCCA
5'selD_MTL_DN	CCAGAGGTTCTGGCATCAGTTTTATCTCAAAATATTATAGAGAAAGAGTGATTGTTTT
3'selD_MTL_DN	CTGCGATCGCGCATGTCTGCAGGCCTCGAGTCTAGTAAGTTGATTTTTCTTCATTT
CRISPR_selD_183	GGTACCTGCTATGGGAGGCAAACCTTGTTTTAGAGCTAGAAATAGCAAGTTAAAATAAGGCTAGTCCGTTATCAACTTGAAAAAGTGGCACCGAGTCGGTGCTTTTTTTCTATGGAGAAATCTAGATCAGCATGATGTCTGACTAGACGCGT
5’Tn916ori	AAGCGGAAGAGCGCCCAATACGCAGGGCCCTAACATCTTCTATTTTTCCCA
3’Tn916ori	TATCTACAATTTTTTTATCCTGCAGGGGGCCCCTAAAGGGAATGTAGATAAATTATTAG
5'selD_comp	CAATTTTTTTATCAGGAAACAGCTATGACCGCGGCCGCACCTAAAATAGGTGAAGCAAC
3'selD_comp	GCCAGTGCCAAGCTTGCATGTCTGCAGGCCTCGAGTTATAAAACTGTAATATATTTTTCC
5' tcdB	TTACATTTTGTTTGGATTGGAGGTC

3' tcdB	AGCAGCTAAATTCCACCTTTCTACC
5' catP 3	ATGGTATTTGAAAAAATTGATAAAAATAG
3' catP 2	TTAACTATTTATCAATTCCTGCAATTCG
5'pyrE 2	GTCCAGTGTTCTGGGGAG
3'pyrE 2	AAAATTTACATTTTTTAAGTAACACTATAAATAATTAAGTTTTTA
5’selD	GAGCTTCCTAAAAATGAAGTAAATATCAATAAACAG
3’selD	TTTTGCTCAAAACAATCACTCTTTCTCTATAATATT
5’COcas9_RT	GTGTAGGATGGGCAGTAATAAC
3’COcas9_RT	CCAAATATTGGATGTCTTTCGTG
5'gRNA_RT	GTTTTAGAGCTAGAAATAGCAAG
3'gRNA_RT	CTGATCTAGATTTCTCCATAG
5'catP_RT	CTTTGCAAGTGTACCTTGTACC
3'catP_RT	GTCAGACTTACACTCAGTCC
oLB70	AAAAGCTTTTGCAACCCACGTCGATCGTGAAGAGCCTAAATATGT GCGCCCAGATAGGGTG
oLB71	CAGATTGTACAAATGTGGTGATAACAGATAAGTCAAATATGGTAA CTTACCTTTCTTTGT
oLB72	CGCAAGTTTCTAATTTCGGTTGGCTCTCGATAGAGGAAAGTGTCT
EBS-universal	CGAAATTA GAAACTTGCGTTCAGTAAAC
oLB76	ATGACAACAGCAGGTGGTTGAGCCGC
oLB77	ATTTGTGCCATTTCTATTGCACC

Uncropped Gel images (labeled)

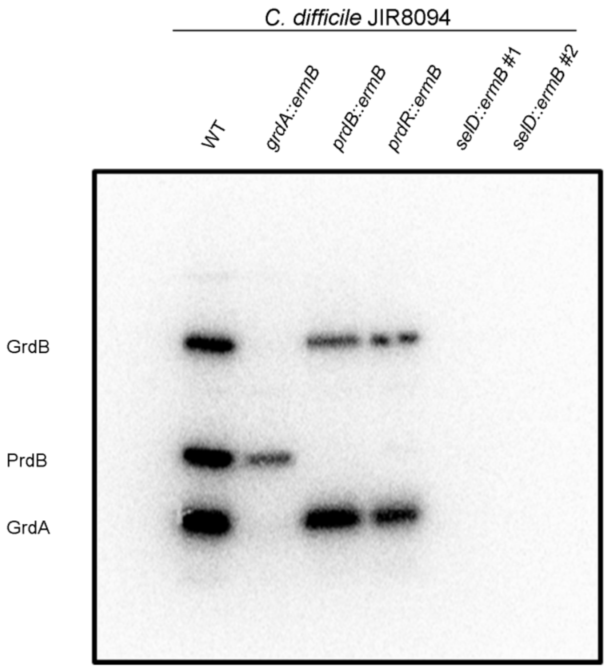

Selenium Labeling (Figure 2). Labels added for clarity, but image is unaltered / uncropped

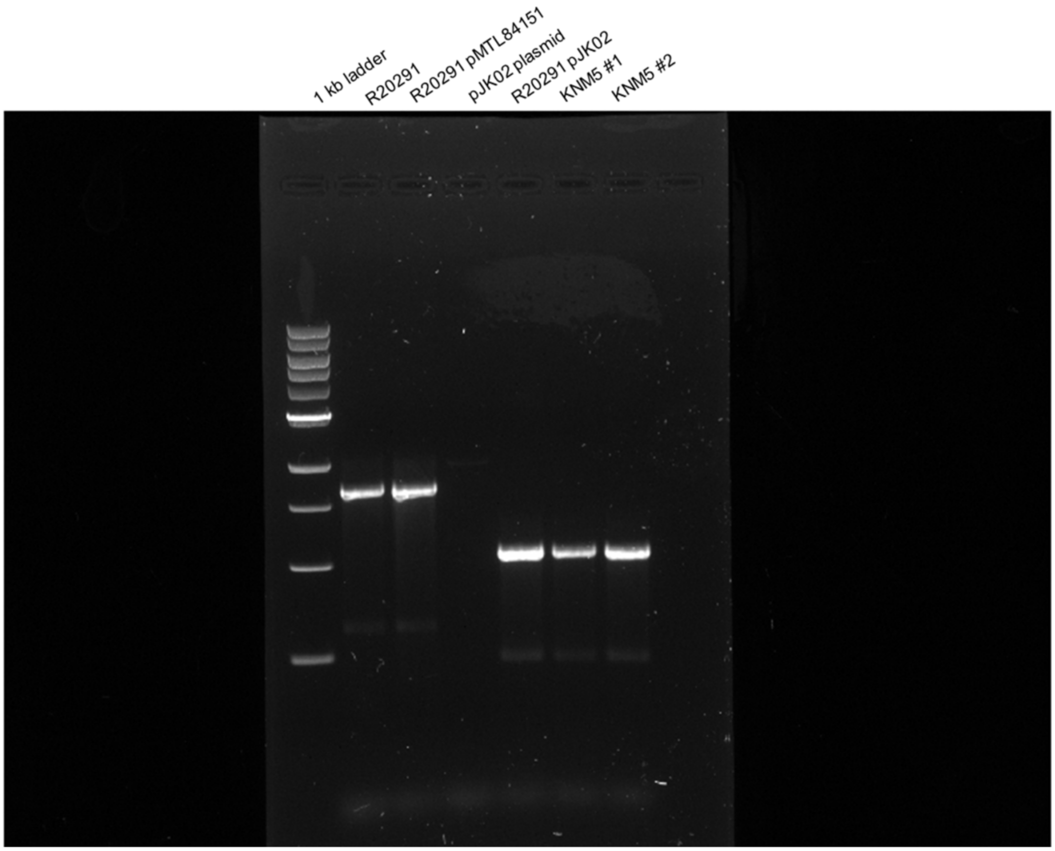

pyrE PCR

PCR of *pyrE* (Figure 3B). Labels added for clarity but the gel images is unaltered / uncropped

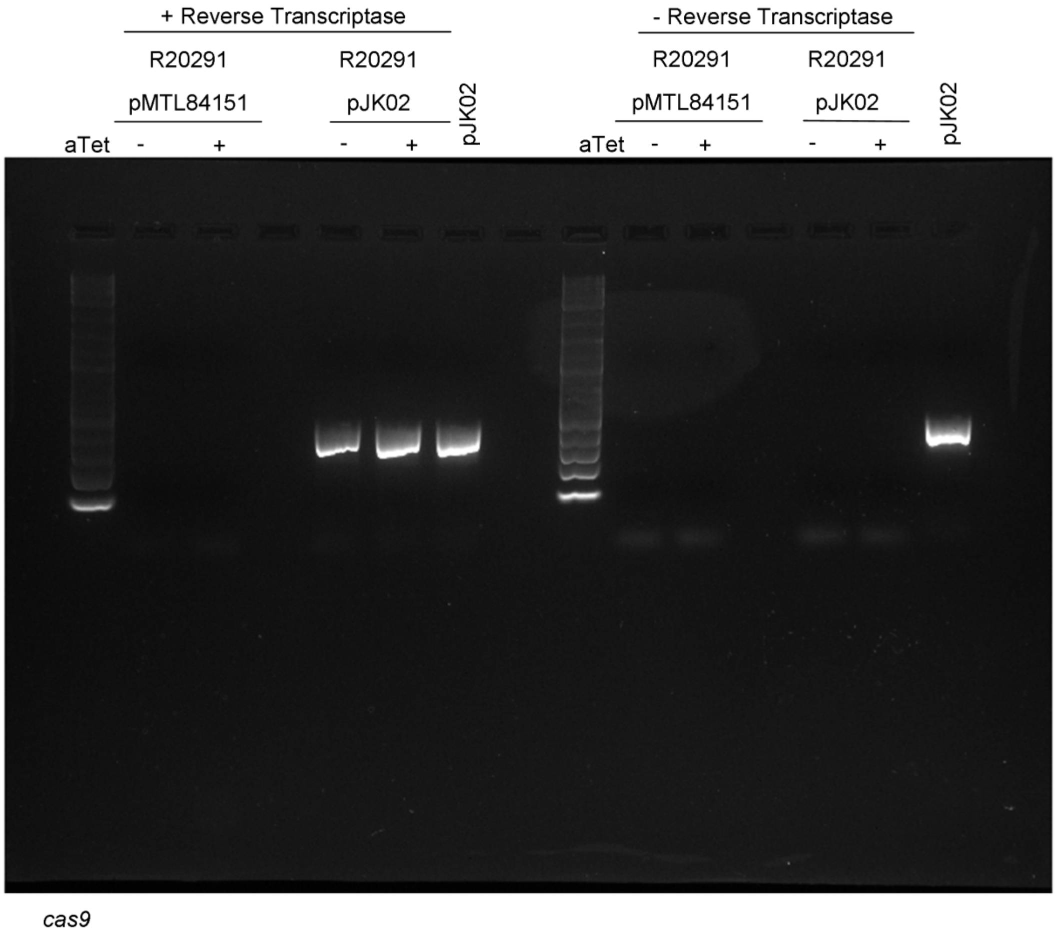

***cas9* RT-PCR (Figure 3E).** Labels added for clarity, but the image is unaltered / uncropped.

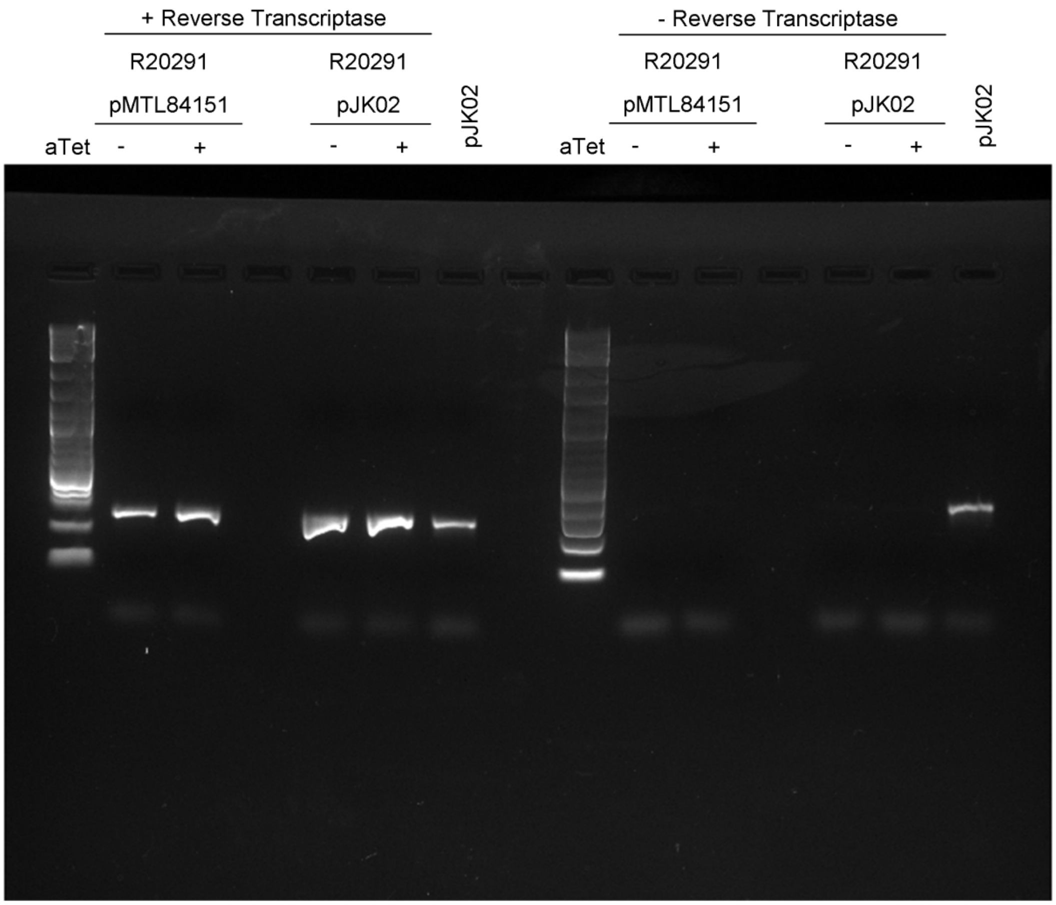

catP

***cat9* RT-PCR (Figure 3E)**. Labels added for clarity, but the image is unaltered / uncropped.

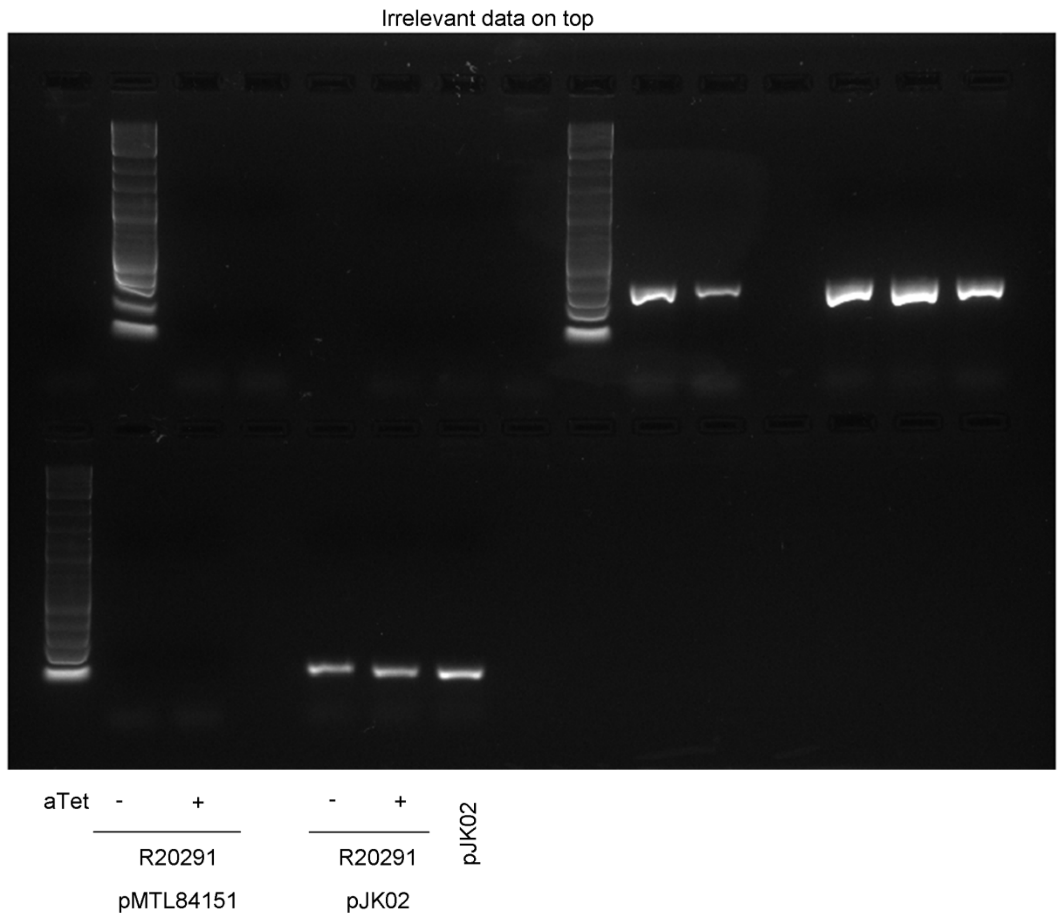

sgRNA RT-PCR (Figure 3E). Plus-reverse transcriptase conditions for the sgRNA RT-PCR on the bottom. Irrelevant data on top. Labels added for clarity, but the image is unaltered / uncropped.

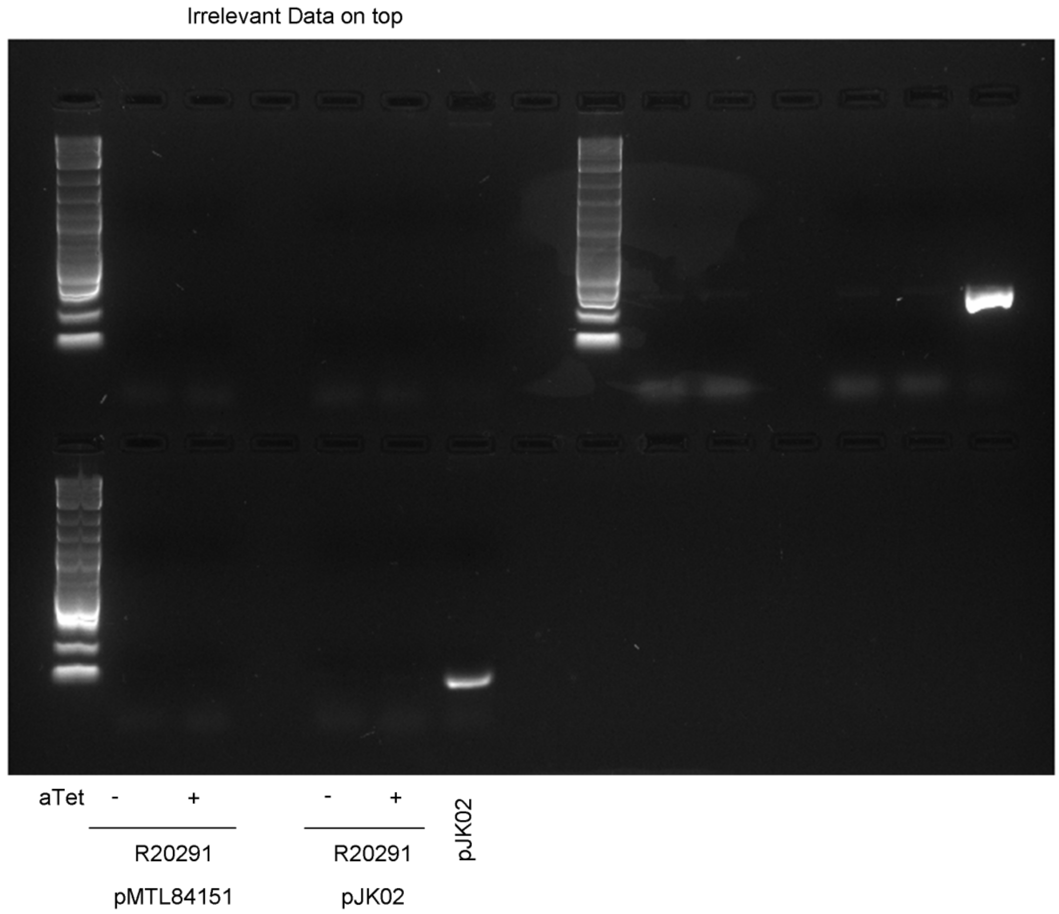

sgRNA RT-PCR (Figure 3E). No-reverse transcriptase conditions for the sgRNA RT-PCR on the bottom. Irrelevant data on top. Labels added for clarity, but the image is unaltered / uncropped
